# Molecular communication relays for dynamic cross-regulation of self-sorting fibrillar self-assemblies

**DOI:** 10.1126/sciadv.abj5827

**Published:** 2021-11-24

**Authors:** Saskia Groeer, Katja Schumann, Sebastian Loescher, Andreas Walther

**Affiliations:** 1 A^3^BMS Lab–Active, Adaptive and Autonomous Bioinspired Materials, Institute for Macromolecular Chemistry, University of Freiburg, Stefan-Meier-Straße 31, 79104 Freiburg, Germany.; 2 Freiburg Materials Research Center (FMF), University of Freiburg, Stefan-Meier-Str. 21, 79104 Freiburg, Germany.; 3 Freiburg Center for Interactive Materials and Bioinspired Technologies (FIT), University of Freiburg, Georges-Köhler-Allee 105, 79110 Freiburg, Germany.; 4 A^3^BMS Lab–Active, Adaptive and Autonomous Bioinspired Materials, Department of Chemistry, University of Mainz, Duesbergweg 10-14, 50447 Mainz, Germany.

## Abstract

Structures in living systems cross-regulate via exchange of molecular information to assemble or disassemble on demand and in a coordinated, signal-triggered fashion. DNA strand displacement (DSD) reaction networks allow rational design of signaling and feedback loops, but combining DSD with structural nanotechnology to achieve self-reconfiguring hierarchical system states is still in its infancy. We introduce modular DSD networks with increasing amounts of regulatory functions, such as negative feedback, signal amplification, and signal thresholding, to cross-regulate the transient polymerization/depolymerization of two self-sorting DNA origami nanofibrils and nanotubes. This is achieved by concatenation of the DSD network with molecular information relays embedded on the origami tips. The two origamis exchange information and display programmable transient states observable by TEM and fluorescence spectroscopy. The programmability on the DSD and the origami level is a viable starting point toward more complex lifelike behavior of colloidal multicomponent systems featuring advanced signal processing functions.

## Teaser

Multicomponent self-assemblies cross-regulate their structures by exchange of molecular information using a metabolic network.

## INTRODUCTION

Complex biological systems can sense and respond to external stimuli and can cross-regulate each other by principles of communication using molecular messengers. This leads to a broad ensemble of spatiotemporally controlled, communicating and self-regulating structures that are orchestrated through signaling systems and feedback loops (*1*, *2*). Such biological reaction networks can be mimicked using chemical reaction networks (CRNs) that show increasingly complex behavior and which have been coupled with self-assembling building blocks to make transient systems of single species (*3*–*6*). However, realizing more elaborate behavior, such as communication and cross-regulation of different entities, remains a challenge (*2*, *7*).

One promising approach toward versatile CRNs is isothermal DNA strand displacement (DSD), in which short complementary domains, termed toeholds, allow for programming of complex reaction networks (*8*) able to sense (*9*), communicate (*10*), and execute simple logic gates (*11*, *12*), as well as compute logic circuits (*13*, *14*). The behavior of DSD circuits is programmable in a straightforward manner because of the Watson-Crick base pairing of DNA and can be predicted using thermodynamic and kinetic models (*12*, *15*, *16*). Several programs (*14*, *17*–*20*), such as Visual DSD (*21*–*23*), have been developed for this purpose. The information processing of DSD circuits complements both biological and chemical systems for a wide range of applications (*24*, *25*). For example, de Greef and co-workers (*10*) showed stable DNA circuits performing versatile functions in protein-based protocell arrays, giving rise to autonomous sensors with cell-like behavior.

Recently, first efforts coupled DSD cascades with structural DNA nanotechnology. For instance, Winfree and co-workers (*26*) used DSD circuits with an amplifier to control the self-assembly (SA) of small DNA tiles, Franco and co-workers (*27*) showed transient DNA fibrils based on a genelet on/off switch with resulting transcriptional control, and Woods *et al.* (*28*) executed Boolean circuits using DNA tiles and a DNA origami seed. In addition, DSD cascades can also be interfaced with adenosine 5′-triphosphate (ATP)–driven CRNs to create previously unknown behavior, such as self-resetting DSD systems, that again can be interfaced with multicomponent structural assembly (*29*). These and other studies (*30*, *31*) give a glimpse on the immense capacity of combining structural and dynamic DNA nanotechnology to pioneer new adaptive and autonomous biosynthetic materials and devices.

Among the multitude of DNA-functional or DNA-active building blocks, DNA origamis, obtained by folding a long DNA scaffold strand into distinct shapes with short staple strands (*32*–*35*), are a prime candidate for integrating DSD circuits with structural DNA nanotechnology because of their capabilities for distinct superstructure formation and precise stoichiometric functionalization. Toehold-mediated strand displacement has been used to reconfigure finite DNA origami superstructures (*36*–*38*), and even first logic gates have been implemented (*39*). We previously showed periodic supracolloidal DNA origami fibrils both by double-stranded DNA (dsDNA) hybridization (*40*) and host/guest interaction with programmable multivalent interactions (*41*, *42*), and introduced reversible DNA origami nanotube formation responding to single-stranded DNA (ssDNA) fuel/antifuel triggers (*43*).

Here, we introduce modular DSD circuits of increasing functionality to program the cross-regulation of two fibril-forming three-dimensional (3D) DNA origami species in an autonomous preprogrammed system via communication relays integrated into the origami species. The DSD circuits control the sorted origami polymerization in time and allow the two distinct origami building blocks to communicate with each other via ssDNA signals. The two DNA species used are (i) 3D DNA origami hollow nanocylinders and (ii) 3D DNA origami nanocuboids, which self-assemble into fibrillar structures depending on the availability of signals. The DSD circuits first realize a negative feedback loop that is subsequently extended by an amplifier module, which enhances the negative feedback, and, lastly, with a thresholding gate to impose additional temporal delays in the signal transduction process. The DSD circuits are based on an in silico design, established with simple ssDNA systems, and subsequently extended to DNA origami. Experimentally, we study the systems using a combination of a in situ Förster resonance energy transfer (FRET) readout and ex situ transmission electron microscopy (TEM). The colloidal level provided by the use of 3D DNA origami will be of interest for future modification with, e.g., enzymes or inorganic nanoparticles. In addition, while communication between species and coupled behavior has been shown on a molecular basis for DNA, this needs to be coupled to changes on the mesoscale to truly mimic biological behavior inspired from biological filaments based on structural proteins, and to transfer these learnings to practical applications in mesoscopic materials at some point. We suggest that this versatile and modular approach for merging structural and dynamic DNA nanotechnology furthers understanding of dynamic soft multicomponent systems while opening up new possibilities for DNA origami superstructures, emulating behavior of biomimetic filaments such as microtubules, which have shown cross-regulation with actin filaments (*44*).

## RESULTS

### Origami and DSD cascade design

The concatenation of DSD reaction networks for the temporally orchestrated and communicative assembly of two distinct colloidal monomer units requires the two assembling species to be (i) distinguishable in imaging methods, (ii) at best amendable to fluorophore functionalization for FRET readout, and (iii) precisely functionalizable to manipulate stoichiometry during exchange of ssDNA signals of the DSD circuit. To address these challenges, we designed two distinct 3D DNA origami: (i) a 3D DNA hollow nanocylinder (abbreviated cylinder) and (ii) a 3D DNA nanocuboid (abbreviated cuboid), which can individually polymerize and depolymerize into hollow nanotubes or solid fibrils, respectively, depending on the availability of ssDNA signals. Compared to 2D origami sheets, these 3D DNA origamis provide a distinct particulate architecture, reminiscent of globular structural proteins as basis of biological filaments, and a higher level of confinement of the participating information relays (2D surfaces in 3D DNA origami versus 1D lines in 2D DNA origami). In addition, facile and fast large area TEM imaging is possible, and the species can be clearly identified.

**Fig. 1.**
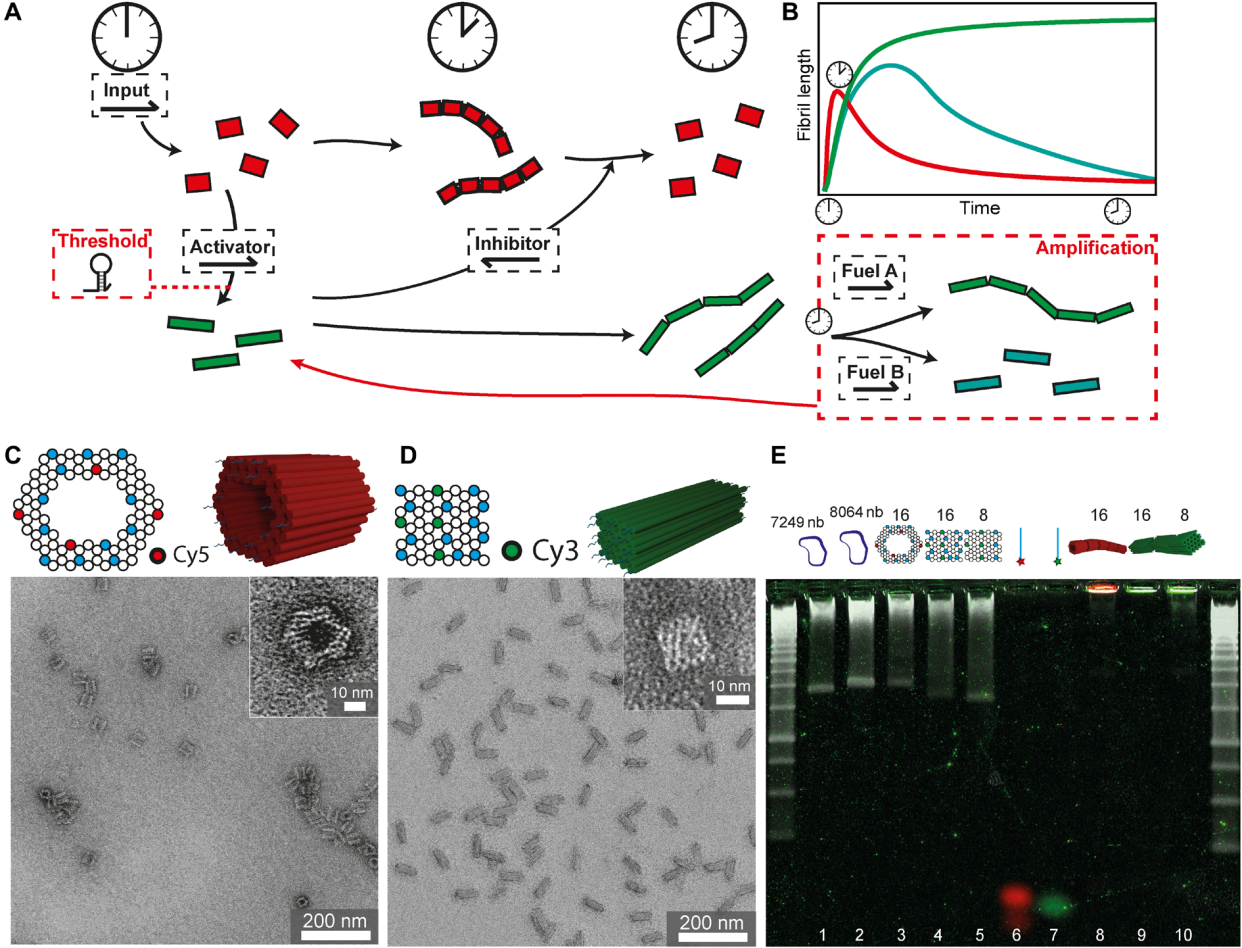
Principle of the DSD circuit controlling DNA origami fibrils and 3D DNA origami characterization. (**A**) Scheme of 3D DNA origami cylinders (red) polymerizing to nanotubes upon input addition, thereby releasing activator, which polymerizes 3D DNA origami cuboids (green) to fibrils. These release inhibitor, which depolymerizes the nanotubes back to single cylinders again. Amplification and threshold modules may tune the feedback signal. (**B**) Schematic fibril lengths of the cylinders (red) and cuboids (green) during the DSD circuit. (**C**) Structure and front view of cylinders with 16 connector positions (blue) including four Cy5-modified positions (red). TEM image of nanocylinders. (**D**) Structure and front view of cuboids with 16 connector positions (blue) including four Cy3-modified positions (green). TEM image of cuboids. (**E**) Overlay of an unstained AGE with its SYBR poststained image. Red and green signals correspond to the Cy5 and Cy3 filter channels in the unstained gel. SYBR poststaining is shown in white with 1-kbp (kilo–base pair) DNA ladders. 1: 7249-nb scaffold; 2: 8064-nb scaffold; 3: cylinders with 16 connectors; 4: cuboids with 16 connectors; 5: cuboids with 8 connectors; 6: all Cy5-modified connectors; 7: all Cy3-modified connectors; 8: nanotubes with 16 connectors; 9: cuboid fibrils with 16 connectors; and 10: cuboid fibrils with 8 connectors.

The basic DSD module governing the communication behavior of these two 3D DNA origami species is sketched in Fig. 1A. An external signal input is given to the premixed systems, which triggers the polymerization of the nanotubes by DSD on the expense of releasing an activator strand for the polymerization of the cuboids. This cuboid activation in turn releases an inhibitor strand during the polymerization of the cylinders, which in a downstream reaction can depolymerize the initially formed nanotube filaments. An amplification module can be added downstream of the polymerized cuboid fibers to (i) strengthen cuboid fibril formation or (ii) break these fibrils into monomers. In both cases, activator is released, which increases the negative feedback for the nanotube disassembly. Alternatively, a temporal thresholding gate can be implemented upstream to delay the cuboid polymerization. The energy provided to execute these autonomous functions is stored in the consecutively used toeholds and hairpin loops, and the system overall approaches equilibrium by following a complex trajectory with metastable areas.

As for the detailed design of the 3D DNA origami (correct folding confirmed by TEM; Fig. 1, C and D), the cylinders are designed with 16 ssDNA connector overhangs with the sequence domain “c1” at one side and with 16 ssDNA connector overhangs with the sequence domain “c1*” at their exact opposite side. All 32 connectors have individual sequences for hybridization to the predetermined positions in the origami scaffold. Four of the c1 connectors are modified with the fluorophore Cy5 marked in red (Fig. 1C), so that quencher-functionalized activator strands, which are initially present to prevent assembly and which are released in the DSD circuit in response to the input strand, quench the Cy5 fluorescence in the cylinders at the start (Fig. 1B). The cuboids similarly contain 16 “c2” and “c2*” connectors at the opposite sides, and four of the c2 connectors are modified with the fluorophore Cy3 (Fig. 1D). Initially, the cuboids are also blocked against polymerization by a quencher-functionalized inhibitor strand, which will be released at intermediate circuit level, providing negative feedback to disassemble the formed cylinder nanotubes. This design, next to the distinction in imaging, allows the use of FRET to monitor the species. All other open ends without connector strands of both origami are passivated by at least nine thymine nucleobases (nb). All DNA sequences used are listed in the Supplementary Materials (tables S1 to S9).

**Fig. 2.**
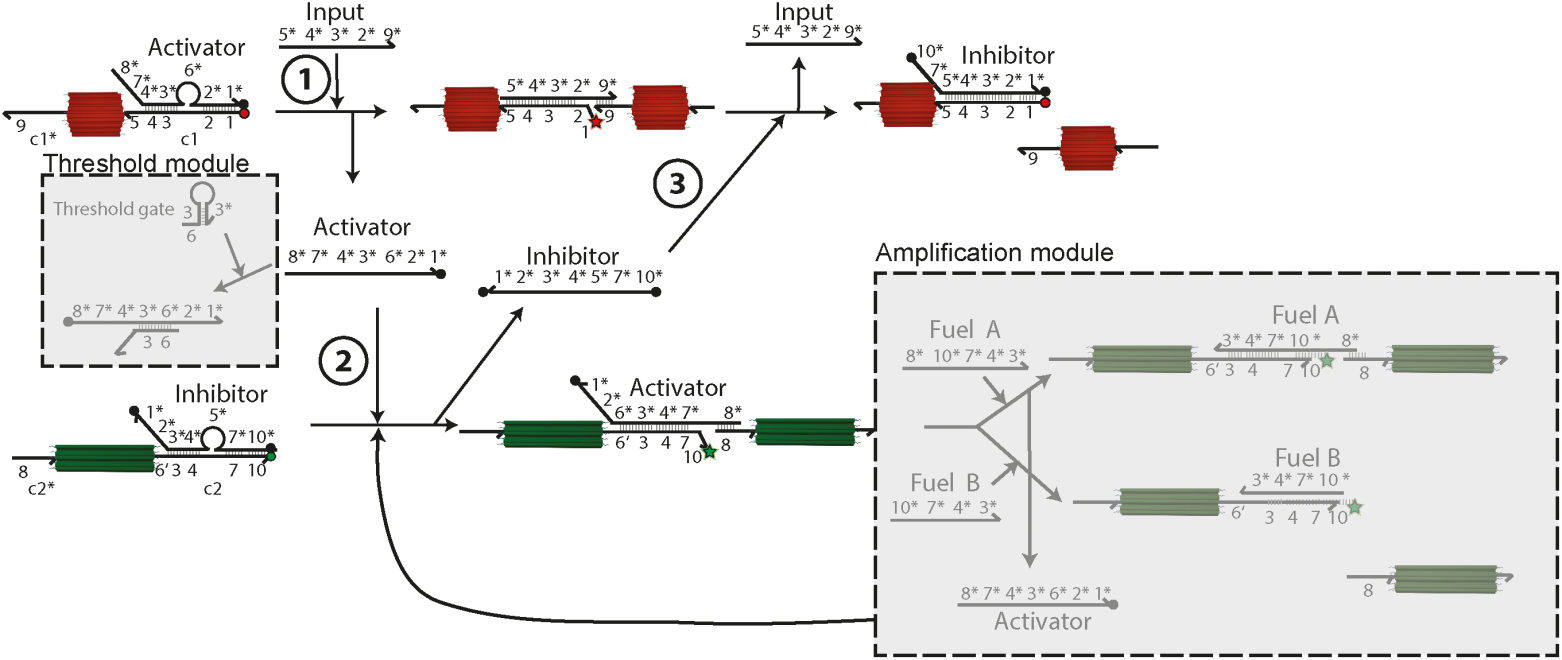
Details of the DSD circuit. Scheme presenting the DSD reactions at the connector ends c1 of the cylinders and c2 of the cuboids with quenchers (black dots) and quenched fluorophores (red/green dots) as well as unquenched fluorophores (red/green stars). The cascade is started by addition of the input DNA strand. Specific sequences are labeled with numbers and their complementary sequence with a “*.” The basic module can be adjusted by adding the amplification module and the threshold module (gray squares).

The origami building blocks were additionally characterized by multicolor AGE (AGE = agarose gel electrophoresis, Fig. 1E). AGE shows single DNA origami bands (lines 3 to 5) that are free of any staple strands after purification by spin filtration. Cylinders are always folded with 16 connectors, whereas the cuboids are prepared with 16 or 8 connectors. These origamis carry the quencher-containing activator and inhibitor strands attached to the fluorescently labeled connectors (c1 and c2) of the cylinder and the cuboid, respectively (see Fig. 2 for sequence labels). The efficiency of the quenching can be understood, as all three origami bands do not show fluorescent signals in the red (Cy5, cylinder) or green (Cy3, cuboid) channel. Only the SYBR staining clearly visualizes the origami bands (white channel) due to spatial separation of the SYBR dye from the quenchers. To confirm the basic SA into fibrils, nanotubes and cuboid fibrils were formed by adding the input or the activator directly to the origami folding mixture instead of the activator and the inhibitor, respectively. In both cases, long self-assembled fibrils are formed that remain in the pocket of the AGE (lanes 8 to 10). In addition, the fluorescence of both fibrils is clearly visible without any SYBR staining. These nanotubes and nanocuboid fibrils are also used as reference for the maximum fluorescence during the time-resolved fluorescence spectroscopy.

The DSD reaction network takes inspiration from earlier work on feedback-driven DSD reaction networks (*10*, *11*, *45*) using adapted sequences and is shown in more detail in Fig. 2 (all ssDNA in table S9). The respective sequence domains are abbreviated by numbers, with complementary domains marked by a *. Sequences labeled with ′, e.g., 6′, carry a mismatched sequence to engineer kinetics. Aside from the toehold length and sequence details, it is important to identify the presence of hairpin loops in activator and inhibitor, which provide additional energetic gains and protect the toeholds by steric constraint until strand release during the operation of the DSD cascade. The cascade is started with the addition of the input. In the basic module, the input displaces the activator (i) via toehold 5 (8 nb) and polymerizes the cylinders to nanotubes. Removal of the activator removes the Cy5/quencher FRET pair and allows for the emergence of Cy5 fluorescence. The released activator displaces the inhibitor from the cuboids (ii) by hybridizing to toehold 6′ (4 nb) with the previous hairpin loop 6*, giving rise to Cy3 fluorescence and polymerization of the cuboids. In turn, the released inhibitor initiates the negative feedback when it displaces the hybridized input (iii) via toehold 1 (9 nb) to depolymerize the nanotubes and quench Cy5 fluorescence. The polymerizing domains 8 and 9 are designed to have similar melting temperatures as previous findings showed that the sequence length of the bridging domains influences the fibril polymerization and length distribution (*43*).

To assure a long lifetime of active cylinders and therefore allow for longer polymerization times of nanotubes, the toehold 6′ at the c2 connector has a mismatch of four bases with the complementary sequence 6* located at the activator. This slows down the displacement of the inhibitor as the rate of DSD is largely influenced by toehold length and sequence mismatch (*46*). This DSD circuit can be further extended by an amplification module and a threshold module, which we will describe later in more detail.

### DSD cascade controlling origami polymerization

**Fig. 3.**
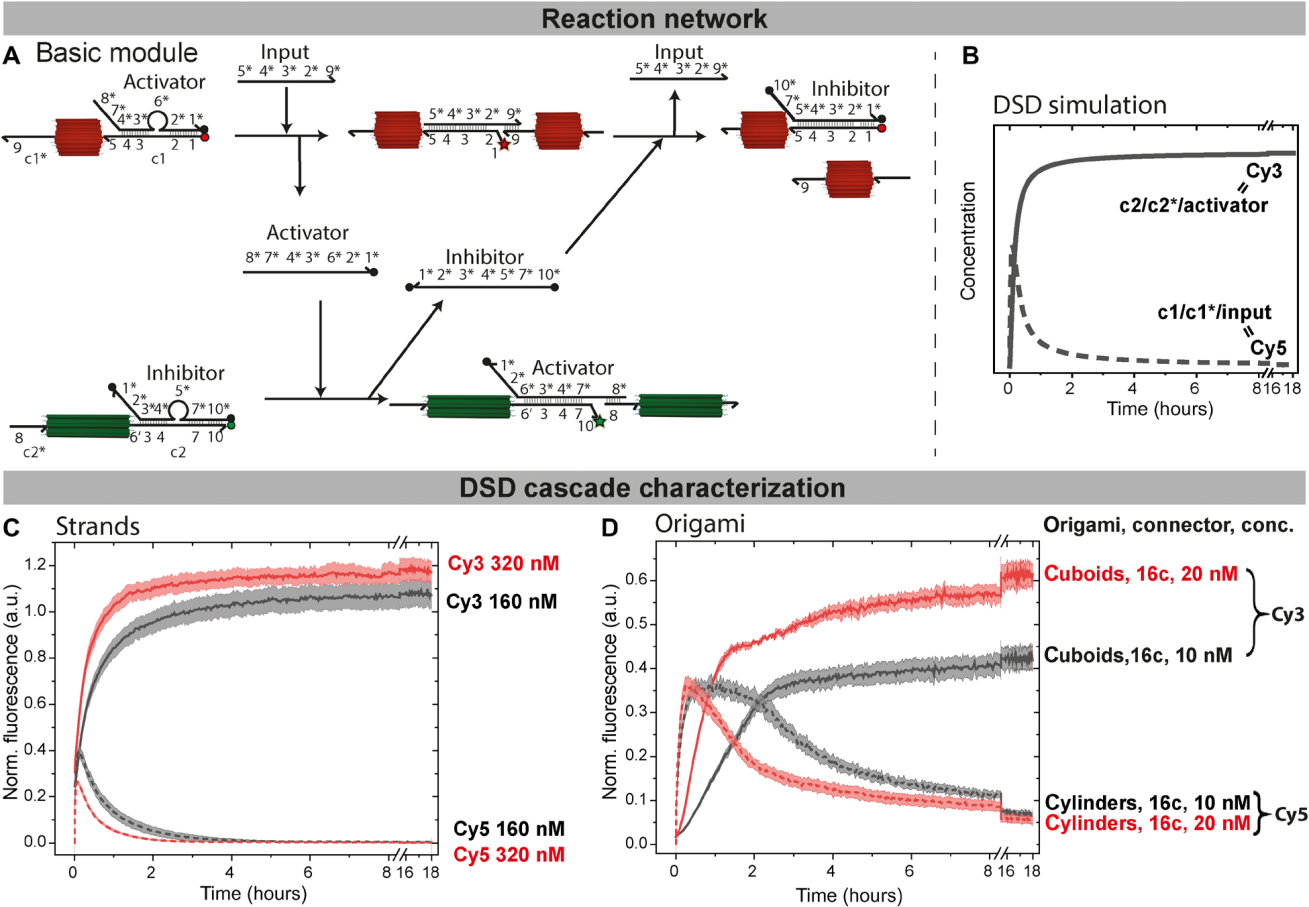
Predictions and FRET verifications of negative DSD feedback loops: From free strands to origami systems. (**A**) DSD reactions of the basic module of the cylinders and the cuboids with quenchers (black dots) and quenched fluorophores (red/green dots) as well as unquenched fluorophores (red/green stars). (**B**) DSD simulation of the cascade predicting the time-resolved behavior of all unquenched Cy3 species (black line; c1/c1*/input) and unquenched Cy5 species (black dotted line; c2/c2*/activator) at 160 and 160 nM input. (**C**) Free strands: Time-resolved normalized fluorescence of Cy3-modified (solid lines; cuboid fibrils) and Cy5-modified (dotted lines; transient nanotubes) DNA strands at 160 nM (black) and 320 nM (red), 1-eq input, respectively. The fluorescence profiles are normalized to a reference experiment containing direct mixtures of either c1/c1*/input or c2/c2*/activator to represent the theoretical maximum fluorescence achievable. The origin of the slightly higher fluorescence of the dynamic system (320 nM, norm. fluor. >1) is not entirely clear but may stem from polarity effects or from the hexadecane layer to prevent evaporation. (**D**) Time-resolved normalized fluorescence of the origami system: 10 nM (black) and 20 nM (red) functionalized cuboids and 10 nM (black, dotted) and 20 nM (red, dotted) functionalized cylinders at 160 or 320 nM input. Note that 16c stands for 16 connector strands; hence, the input is stochiometric. Normalization is done by comparing the fluorescence to that of origami gently polymerized with the relevant strands during a folding/temperature annealing. All fluorescence measurements are an average of 2; the shaded area is the SD. a.u., arbitrary units.

Simulations of the basic module of the DSD circuit (Fig. 3A) using Visual DSD (*21*) confirm the presence of transient species and the principal operation of the circuit (Fig. 3B). The codes used for all simulations are listed in the appendix in notes S1 to S3. Experimentally, we first tested the basic module using the connector strands only—not yet incorporated into the origami—to confirm the simulations and validate the FRET assay. The complexes of c1/activator as well as c2/inhibitor were preannealed in FoB5 buffer [5 mM tris, 1 mM EDTA, 5 mM NaCl, and 5 mM MgCl_2_ (pH 7.2)] by heating to 95°C and cooling down to 4°C within 20 min. The complexes were then mixed with the connectors c1* and c2* at a concentration of 160 nM for each species while increasing the salinity to 10 mM MgCl_2_. The reaction was initiated by addition of input, and the fluorescence of Cy3 and Cy5 was recorded for 18 hours at 37°C. The fluorescence profiles are normalized to a reference experiment containing direct mixtures of either c1/c1*/input or c2/c2*/activator to represent the theoretical maximum fluorescence achievable. In the dynamic systems, the maximum Cy5 fluorescence, corresponding to the three-partner complex of c1/c1*/input, is reached within 10 min (Fig. 3C) for a mixture of 160 nM. Afterward, the fluorescence decreases because of the quenching by the inhibitor and the formation of the final c1/inhibitor complex after ca. 2 hours. Concurrently, the Cy3 fluorescence increases for 2 hours because of the removal of the inhibitor from c2/inhibitor and the formation of the final c2/c2*/activator complex. Afterward, it also levels off and stays constant. Consequently, after 2 hours, the DSD circuit is completed and the inhibitor has displaced the transiently hybridized input completely.

At higher concentrations of 320 nM (Fig. 3C), the increase in Cy3 fluorescence is faster and the maximum of Cy5 fluorescence is reduced, because the negative feedback sets in more quickly. The concentrations used for the ssDNA experiments without any origami correspond to mixtures of 10 and 20 nM origami with 16 connectors, respectively.

Last, the DSD concept was extended to the communication of cylinders and cuboids functionalized with activator and inhibitor, respectively. We first performed a leakage check of the network in the absence of the input for mixtures of 10 nM of both origamis with 16 connectors (fig. S1). Such leakage in DSD cascades is a common phenomenon and is caused by errors during DNA synthesis and blunt end strand exchange (*8*, *47*). In our system, because there is no increase in Cy5 fluorescence, and only a minor Cy3 fluorescence increase over 18 hours, the overall network can be considered free of any major leakage and ready for implementation.

The full implementation of the DSD reaction network was achieved by adding 1 eq of input versus connector strands for mixtures of 10 nM of both origami with 16 connectors (Fig. 3D, black lines). The maximum Cy5 fluorescence, normalized to the fluorescence of origami polymerized gently during temperature annealing as described previously, is reached within 30 min and decreases after about 2 hours, while, similar as above, the Cy3 fluorescence increases to a plateau in the same time frame (Cy3 fluorescence is also normalized to the origami gently polymerized during temperature annealing). The low standard deviations (SDs) of the duplicate experiments confirm good reproducibility. Yet, it is interesting to observe that the Cy3 fluorescence does not return to 100%. This is however not caused by stochiometric mismatches of strands (because the SD of duplicate measurements could not be kept low in case of excessive pipetting errors) but by the fact that Cy3 fluorescence strongly depends on the DNA microenvironment and conformation. We suggest that the isothermal DSD reaction can lead to some metastable traps at the crowded DNA origami surface hindering fluorescence emission compared to gentle annealing using a slow folding temperature ramp. Intensity losses of greater that 50% have been for instance described already for comparably simple DNA structures (*48*), and we will see below that releasing the conformational restraints of the Cy3 dye using a specific downstream module (fuel B, amplification module; Fig. 5G) brings the fluorescence close to 100%. In terms of kinetics, clearly, a reduced reaction rate is found compared to the DSD cascades based on the free strands (Fig. 3C). This temporal delay is an architectural effect provided by the crowded regions of the origami tips, which are kinetically less accessible compared to strands in free solutions. In addition, it is caused by the fact that diffusion of the origami (and their fibrils, as seen below) is also much slower, yielding reduced reaction kinetics (*49*, *50*). Again, a higher reaction rate can be achieved by increasing the origami concentration to 20 nM each (Fig. 3D, red lines). Nonetheless, the longer lifetimes of the intermediate fibril structures at lower origami concentration of 10 nM are favorable for imaging purposes; thus, 10 nM was chosen for future experiments.

**Fig. 4.**
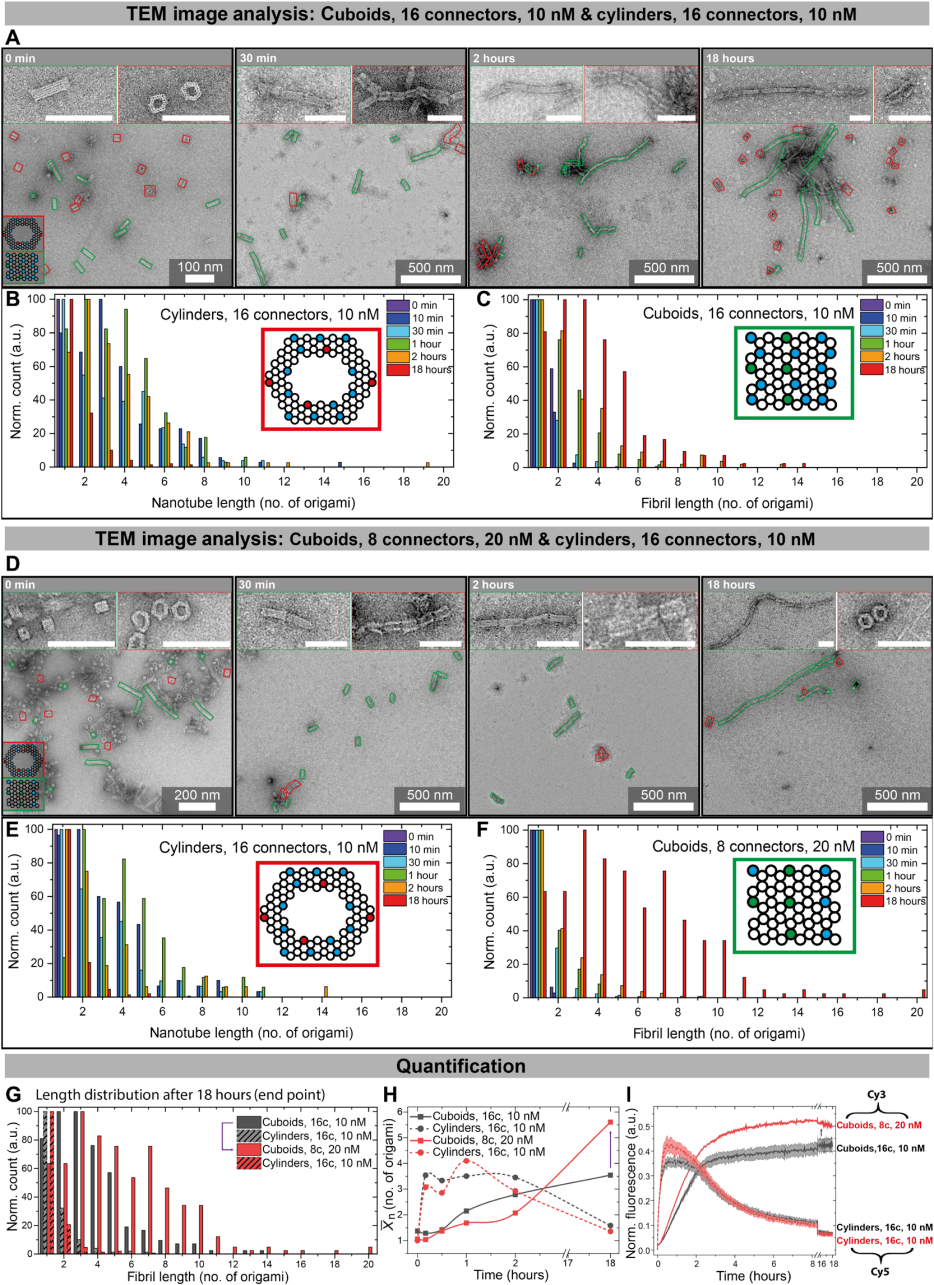
Time-dependent characterization of the fibril length distributions via statistical TEM analysis. (**A**) Time-lapse TEM images of origami polymerization for 10 nM cuboids (green, 16c) and 10 nM cylinders (red, 16c). (**B** and **C**) Statistical TEM image analysis of (B) cylinder nanotube and (C) cuboid fibril lengths using 16c and 10 nM for both origami. The most abundant species at each time point is normalized to 100. (**D**) Time-lapse TEM images of origami polymerization for 20 nM cuboids (green, 8c) and 10 nM cylinders (red, 16c). (**E** and **F**) Statistical TEM image analysis of (E) nanotube and (F) fibril lengths using 20 nM cuboids (8c) and 10 nM cylinders (16c). The most abundant species at each time point is normalized to 100. (**G**) Statistical TEM image analysis of fibril lengths after 18 hours for both systems, comparing samples of (B), (C), (E), and (F) at 18 hours. (**H**) Further quantification of (B), (C), (E), and (F) by plotting ${\bar{X}}_{n}$ for the DSD cascade with 10 nM cuboids (16c, black) and with 20 nM cuboids (8c, red). Cylinders in both cases at 10 nM with 16c. Lines are Bezier curves. (**I**) Time-resolved normalized fluorescence of Cy3- and Cy5-modified cuboids and cylinders. All fluorescence measurements are an average of 2; the shaded area is the SD. Colored lines in TEM images are added as guide to the eye for identifying both origami types. Scale bars, 100 nm.

TEM was used to visualize how the fluorescence response correlates with structural changes in the fibrillating origami for the standard mixture of 10 nM origami at 16 connector strands (Fig. 4A). The longest nanotubes are found between 30 min and 2 hours (Fig. 4, B and H) and reach a maximum length of up to 19 origamis per nanotube with a number average degree of polymerization ${\bar{X}}_{n}$ of 3.5. This matches the plateau previously seen in the fluorescence measurements of Cy5 (Fig. 3D). After 18 hours, the negative feedback invoked by the cuboid polymerization depolymerizes the nanotubes to mostly monomers; only a few dimers and trimers remain (Fig. 4G). This also agrees with the fluorescence data, as a slight Cy5 fluorescence is observed even after 18 hours (Fig. 4I). As for the communicating cuboids, some dimers are observed even before input addition because of base stacking effects (Fig. 4A). After input addition, the fibril length quickly increases within the first 2 hours (Fig. 4, C and H) and levels off afterward, again agreeing with observations from the fluorescence data. The fibrils grow up to 14 cuboids per fibril at an ${\bar{X}}_{n}$ of 3.5. Because this polymerization is similar to a step growth polymerization, the observation of a low ${\bar{X}}_{n}$ is expected in a number-weighted distribution analysis.

When mixing 20 nM cuboids with only eight connectors together with 10 nM of cylinders with 16 connectors [to maintain the same overall stoichiometry of c1 and c2 (160 nM)], distinct differences in the distributions can be observed in Fig. 4 (D to F). While the overall trend is similar as above, a direct comparison of the cuboid lengths in Fig. 4G reveals that the cuboids with eight connectors reach higher lengths of up to 20 cuboids with a ${\bar{X}}_{n}$ of 5.6 after 18 hours (end point), thus increasing ${\bar{X}}_{n}$ by 2.1 (Fig. 4H).

It is interesting to realize that longer cuboid fibrils are formed despite the fact that a smaller number of interacting connectors (hence lower multivalency) is present on the cuboid, which has been previously shown to influence fibril polymerization (*41*–*43*). The fluorescence data show a quicker increase in Cy3 fluorescence for 20 nM cuboids than for 10 nM cuboids (Fig. 4I). This increases the speed with which the bridging domain 8 is available for cuboid polymerization. Hence, it becomes clear that, in particular, the cuboid polymerization kinetics are accelerated because of the doubling of the cuboid concentration and concomitant increase in effective collisions per time unit. This leads to the observation of longer cuboid fibrils at higher cuboid concentration, even for lower amounts of interacting sites at the tips in the same time frame.

To sum up, the whole DSD negative feedback network with transient cylinder nanotube formation coupled to cuboid nanofibril polymerization can be followed closely by both in situ fluorescence spectroscopy as well as by visualization of the resulting DNA superstructures by ex situ TEM. The communication between both building blocks, which self-sort into distinct and separate fibrils, yields two simultaneous and coupled polymerizations and an autonomous system for dynamic, transient nanotubes.

### Signal amplification modules to enhance the negative feedback and regulate the cuboid end point state

One large benefit of using DNA as a regulatory component in CRNs is the facile adjustment and expansion of a given CRN owing to the exact and orthogonal programming of DNA. We first focus on the introduction of an amplification module, which has two variations, A and B, that have an influence on promoting robustness of the negative feedback as well as allow to regulate the final state of the cuboid fibril assembly.

**Fig. 5.**
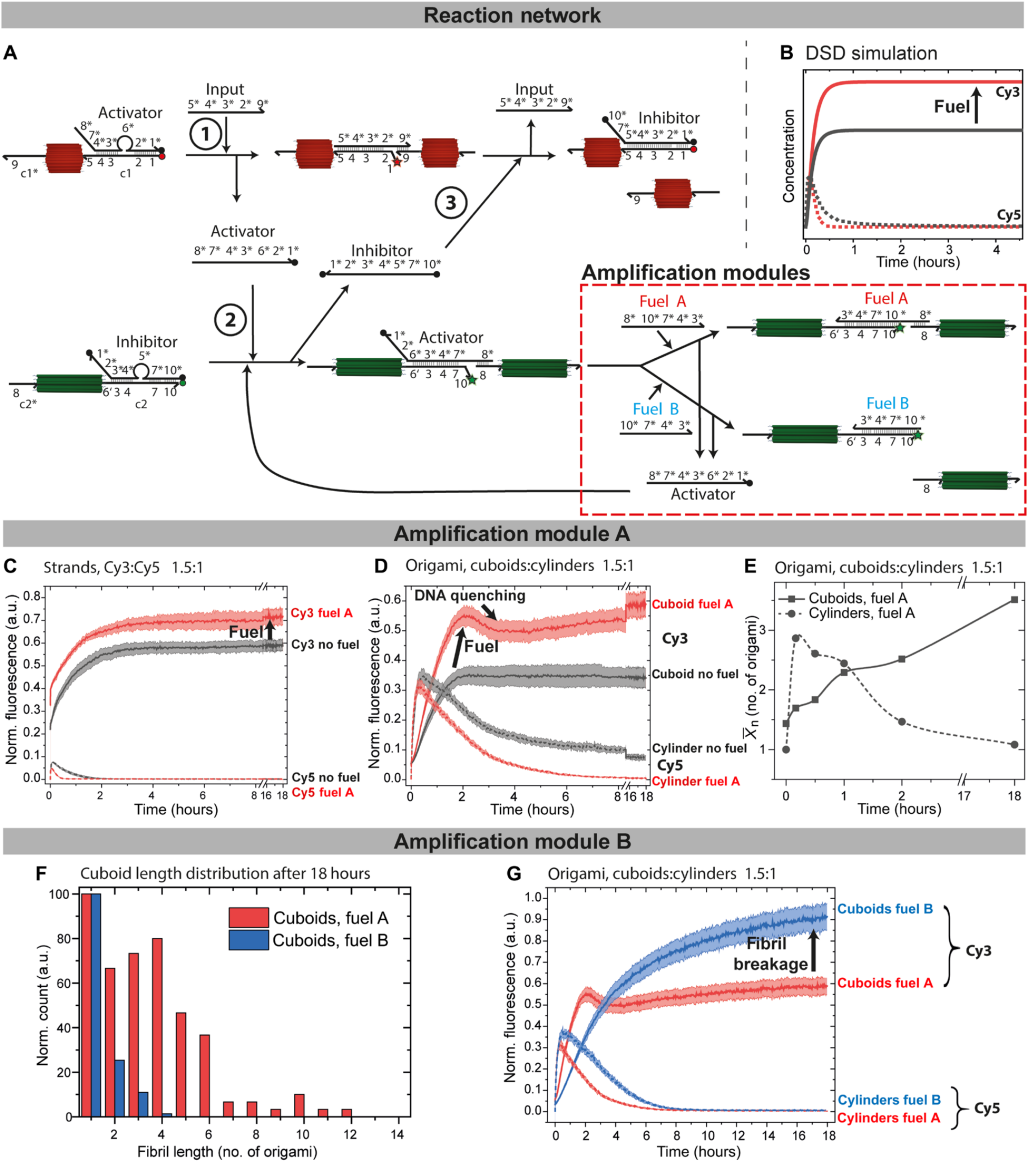
Signal amplification modules and end point regulation of the cuboid assembly. (**A**) Scheme of the DSD cascade with the amplification modules. Fuel A displaces the activator while keeping cuboid fibrils intact. Fuel B depolymerizes the cuboid fibrils. (**B** and **C**) DSD simulation and experimental implementation of the fueled DSD circuit with (red) or without (black) fuel A. DSD predicts the behavior of the unquenched Cy3 species (solid lines) and unquenched Cy5 species (dotted lines), while the time-resolved normalized fluorescence confirms the behavior. c1/activator, c1*, input at 160 nM, inhibitor/c2, c2*, fuel A at 240 nM. (**D**) Time-resolved normalized fluorescence of the origami system: 15 nM functionalized cuboids (16c) and 10 nM functionalized cylinders (16c) at 160 nM input in the presence of 240 nM fuel A (red) and without fuel (black). (**E**) ${\bar{X}}_{n}$ for the DSD cascade with fuel A. Lines are Bezier curves. (**F**) Statistical TEM image analysis of cuboid fibril length distribution after 18 hours with 80 nM fuel A (red) or 480 nM fuel B (blue) using 15 nM cuboids and 10 nM cylinders. (**G**) Time-resolved normalized fluorescence comparing the effect of fuel A versus fuel B [conditions as in (D)]. All fluorescence measurements are an average of at least 2; the shaded area is the SD.

In variation A, the additionally present fuel A displaces the bridging activator from the c2/c2*/activator complex using toehold 10 (7 nb; Fig. 5A). This does not change the fibril connectivity but replenishes the activator for signal amplification from the upstream inhibitor-modified cuboid. This upstream action both activates more cuboids more quickly for polymerization and accelerates nanotube breakage. On a system’s level, this additionally means that cuboids with the c2/inhibitor complex can be present in excess and form fibrils even if the activator concentration is low. Hence, we elucidate the fuel-containing system for a 1.5/1 ratio of c2/inhibitor (cuboids) to c1/activator (cylinders) for fuel A. The excess 0.5-eq inhibitor can only be displaced if activator is replenished by fuel A. Experimentally, and in the DSD simulations, this signal amplification is confirmed by an increase in Cy3 fluorescence compared to the same mixture without fuel A. In addition, the quenching of Cy5 is faster in the presence of fuel A (Fig. 5, B and C; all simulated fuel concentrations in fig. S2).

When applying this to the origami system, the use of fuel A increases the amount of cuboid fibrils, while it reduces the nanotube lifetime, granting further tunability to the system (Fig. 5A). For instance, using 15 nM of cuboids with 16 connectors and 10 nM of cylinders with 16 connectors in the presence of 160 nM of fuel A leads to an increase in Cy3 fluorescence related to the amplification (Fig. 5D). After a fast increase, Cy3 is slightly quenched after 2 hours, which can be attributed to the quenching effect the DNA has in this configuration with fuel A (*51*) because the fluorophore-modified end is located inside a three-strand complex of c2/c2*/fuel A [note that this does not occur for free DNA strands (Fig. 5C) (*52*); further details on Cy3 fluorescence in different DNA structures can be found here (*53*)]. When paired with activator, the fluorophore-modified end is unhybridized and not in direct proximity to DNA, therefore showing higher fluorescence. The comparison to the Cy5 fluorescence in the origami-integrated system shows that the lifetime of the nanotubes is shortened in the presence of fuel A, as predicted by both the simulation and reference measurements without origami (Fig. 5D). Moreover, the use of fuel A quenches the Cy5 fluorescence during the negative feedback more completely, and after 8 hours, Cy5 is fully quenched. Consequently, by releasing an excess of inhibitor during signal amplification, the negative feedback signal is intensified, and the network has gained functional robustness.

The TEM characterization of the system aligns with the fluorescence measurements. Representative TEM images are shown in fig. S3. The nanotube lengths show a slightly reduced maximum nanotube length of 14 instead of 19 cylinders (without the module) with a ${\bar{X}}_{n}$ of 2.9 at 30 min, after which ${\bar{X}}_{n}$ decreases again. This confirms an earlier suppression of the cylinder polymerization and a decrease in the nanotube lifetime (Figs. 4H and 5E; more details in fig. S3). The excess of released inhibitor and the thereby enhanced negative feedback cause a complete depolymerization of nanotubes down to monomers with a ${\bar{X}}_{n}$ of 1.1. The communicating cuboid fibril lengths closely resemble the lengths previously observed for 10 nM and 16 connectors without fuel A with a ${\bar{X}}_{n}$ of 3.5. The cuboid polymerization proceeds very similar to the basic module, yet at a 1.5 excess of cuboids. Hence, in total, more cuboids are polymerized by replenishing activator without the need of additional input, thereby manifesting signal amplification also on the cuboid side with enhanced cuboid polymerization.

The second amplification module B (fuel B) is different to module A in the sense that, while fuel B is also able to displace the activator using the same toehold 10, it does no longer bridge the cuboids in the fibril as it lacks the necessary sequence 8* for hybridization with c2* (Fig. 5A). TEM image analysis after 18 hours of the DSD cascade shows depolymerization and mostly single cuboids and a low ${\bar{X}}_{n}$ (Fig. 5F and fig. S3). Consequently, the transient nature of the self-assembling species is now extended from the cylinder to the cuboids, and their behavior is synchronized regarding the transient nature of the self-assembling fibrils. When comparing the fluorescence data of the circuit kinetics of both amplification modules in situ, the quenching of Cy3 by DNA structures is less pronounced when no c2* is hybridized (fibrils broken) (Fig. 5G). Use of fuel B results in a longer lifetime of the Cy5 fluorescence and slower increase in Cy3 fluorescence compared with fuel A, which is even more underscored by the fact that we used an intentional excess of fuel B compared with fuel A for this comparison. This suggests that connector c2* can act as an additional toehold for fuel A if the activator is only partly hybridized and hence increases the reaction rate of activator displacement with fuel A if the origamis are already in vicinity. The fluorescence intensity at the end of the fuel B–enhanced DSD cascade shows close to 100% of the maximum expected value because the Cy3 dye is conformationally relatively unconstrained in this case.

### Delay of negative feedback with a threshold module

**Fig. 6.**
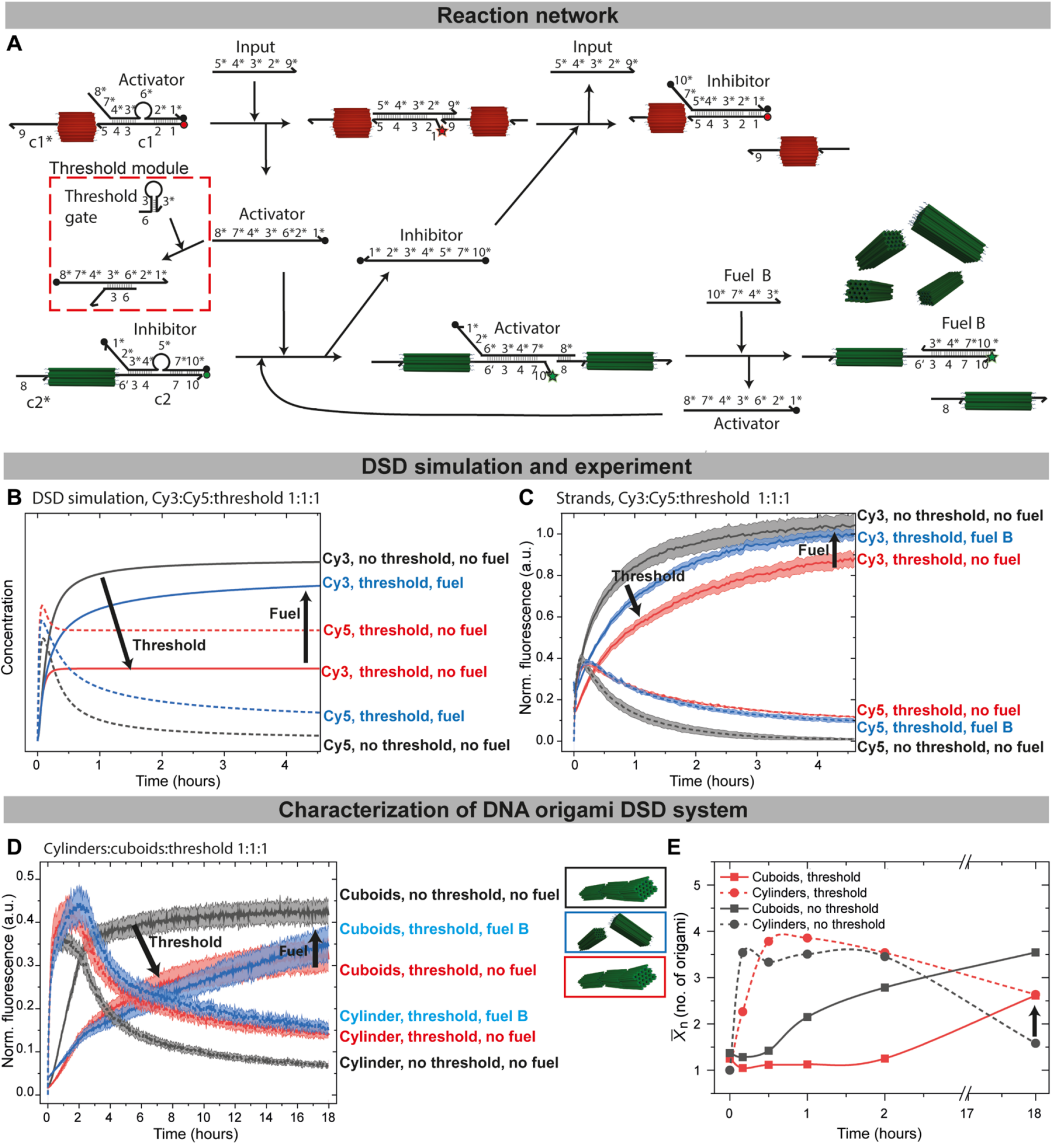
Feedback delay by the threshold module. (**A**) Scheme of the feedback delay by use of a threshold module. The activator reacts faster with toehold 6 of the threshold gate than with toehold 6′ of connector c2 due to a 4-nb mismatch. (**B**) DSD simulations in the presence or the absence of 160 nM threshold gate and 160 nM fuel B: c1/activator, c1*, c2/inhibitor, and c2* at 160 nM. Unquenched Cy3 and Cy5 species are plotted. (**C**) Time-resolved normalized fluorescence of free strands [conditions as in (B)]. (**D**) Time-resolved normalized fluorescence of 10 nM functionalized cuboid and cylinder in the presence or the absence of 160 nM threshold and 160 nM fuel B. Input = 160 nM. (**E**) ${\bar{X}}_{n}$ for the negative feedback loop with 160 nM threshold (red) and without threshold (black). All fluorescence measurements are an average of at least 2; the shaded area is the SD.

The nanotube length distribution, ${\bar{X}}_{n}$, depends both on the fraction of hybridized input and the active polymerization lifetime because polymerization on the colloidal scale is a comparably slow process (*43*). Therefore, we hypothesized that a delay in the negative feedback could lead to longer nanotube lifetimes and thus a higher ${\bar{X}}_{n}$. To tackle this challenge, we embedded a threshold module (*11*) just downstream of the input-to-activator conversion to impose a delay (Fig. 6A). The threshold gate is a hairpin loop with a toehold 6 (8 nb) completely complementary to 6* of the activator and was iteratively improved by DSD simulations. The selected threshold gate sequence leads to a faster reaction compared to the displacement of the inhibitor from c2, because the 6′ mismatches 6* by 4 nb, slowing down the reaction. It largely acts as a competitive sink, with little chance of downstream reaction as identified by Visual DSD (Fig. 6B, red lines).

This module can be combined with the amplification module, as exemplified here for the module B. Addition of fuel B helps to more efficiently quench Cy5 fluorescence (depolymerized nanotubes) by enforced downstream reaction and release of more inhibitor forming inhibitor/c1. It also leads to higher Cy3 fluorescence by self-promotion of fuel B/c2 complex formation, which is confirmed in silico (Fig. 6B, blue lines).

Again, first reference measurements without any origami were performed (Fig. 6C). As expected, the addition of the input to an equimolar mixture of all components (activator/c1, inhibitor/c2, and threshold gate at 160 nM each) leads to a delayed negative feedback. This is most evident from the slower increase in Cy3 fluorescence, the higher Cy5 fluorescence in the transient state, and by the delayed decrease in the Cy5 fluorescence, which can be explained by a partial or intermediate scavenging of the activator by the threshold gate (*54*). Some of this activator signal can be restored by use of fuel B, replenishing the activator to release more inhibitor for the negative feedback.

When transferring the threshold principle to the origami with cylinders:cuboids:threshold = 1:1:1, the threshold delays the Cy3 fluorescence increase (Fig. 6D), and the maximum Cy3 fluorescence at the end is also lower. Therefore, this observation is in line with simulated predictions. The recovery of the Cy3 fluorescence with fuel B is less pronounced compared to simulations, which is likely due to the reduced reaction rates found in origami. Similarly, the Cy5 fluorescence decays more slowly and not to the same level in the presence of the threshold, which is an indication for a better nanotube polymerization.

The delay of the negative feedback and hence a longer nanotube lifetime results in higher nanotube length according to TEM image analysis (Fig. 6E, representative TEM images in fig. S4). The length distribution is shifted toward higher lengths overall with a ${\bar{X}}_{n}$ of 4.2 after 30 min compared to the previously observed 3.5 in the DSD cascade without threshold. In addition, the depolymerization of the nanotubes is notably delayed in the presence of the Threshold and better suppressed (end point; Fig. 6E). After 18 hours, the cuboid fibrils only reach a reduced ${\bar{X}}_{n}$ of 2.6 compared to a ${\bar{X}}_{n}$ of 3.5 without threshold (both in the absence of fuel B); many monomers remain. This arises from the scavenging of the activator by the threshold gate, as predicted by DSD simulation and measured in fluorescence spectroscopy.

## DISCUSSION

We introduced the first example for autonomous cross-regulation of self-sorting multicomponent self-assembling systems by concatenating DSD scenarios with molecular information relays embedded in the self-assembling species. The approach merges structural DNA nanotechnology in the form of precisely programmable 3D DNA origami with a DSD-CRN to precisely orchestrate the reconfiguration of superstructures proceeding via an intermediate 3D DNA origami colloid level. The colloidal 3D nature facilitates distinction in microscopy and mimics globular protein structures for biomimetic filament design. Even though on a rudimentary level, the fibrillar structures mimic some behavior of the living cytoskeleton, where various fibrillar structures such as microtubules and actin filaments cross-regulate (*44*) and are organized temporally.

Because of the versatility of the DSD system, the basic negative feedback module could be extended with amplification and thresholding modules, and the in silico optimization provides useful guidance for experimental design and analysis. The amplification module further allows to change the end point state (fibril or monomer) for the origami cuboids to synchronize the transient behavior with the origami cylinder nanotubes. Gratifyingly, both origami building blocks are easily distinguishable by TEM, allowing to quantify the respective fibril length distribution throughout the course of the DSD circuit, a distinct advantage of this system compared to DNA tile–based approaches. Next to the temporal features of the DSD circuit, both the origami concentration and the connector density influence the fibril lengths. Compared to pure strands in solution, the origami system experiences delays in the operation of the circuit because of the spatial confinement at the origami tips; yet, they continue to operate smoothly despite the higher level of confinement and higher dimensionality of the spatially immobilized sequences: 2D in 3D DNA origami versus 1D in 2D sheet origami or molecular scale for DNA tiles.

The DSD circuit presented herein provides a viable starting point to further extend the behavior by, for instance, including catalytic DSD cycles, ATP-driven DSD cycles, or even incorporate additional external switches and signal processors (*29*). First examples on DNA tile–based fibrils have shown fascinating approaches for redox (*55*) or pH (*56*) controlled polymerization, and combining these approaches both with DNA origami as colloidal monomers and with DSD computing is promising for sensing and even biomedical applications. In addition, the elaborate design of the multivalency patterns at the interacting tips can offer broad opportunities including pattern recognition (*42*). We also suggest that the control principles should be applicable to other colloidal structures, which can allow, at the least, the organization of relevant photonic effects in structured matter (*57*).

## MATERIALS AND METHODS

### Reagents

Agarose gel was received from AppliChem. SYBR was ordered from Fisher Scientific. MgCl_2,_ NaCl, boric acid, hexadecane, magnesium acetate, and tris were obtained from Sigma-Aldrich. EDTA was purchased from Carl Roth. Carbon film 300 mesh copper grids and uranyl acetate (>98%) were bought at EMS. Milli-Q water with a conductivity below 0.055 mS/cm was used during all experiments.

### Oligonucleotides

Scaffolds (70,249 and 8064 nb) were purchased from tilibit, and DNA strands were purchased from IDT and IBA Lifesciences. All DNA strands used for CRNs were bought in high-performance liquid chromatography grade. Stock solutions were prepared by dissolving the lyophilized DNA in salt-free TE [10 mM tris and 1 mM EDTA (pH 8)] buffer. Iowa Black was chosen as quencher.

### Methods

#### UV-visible spectroscopy

Ultraviolet (UV)–visible measurements were conducted on an AnalytikJena ScanDrop 250 using a Hellma TrayCell with a path length of 1 mm. DNA concentrations were calculated using the absorption at 260 nM and the calculated extinction coefficient for the respective DNA strand. DNA origami concentrations were measured using the estimated conversion of absorbance A_260_ of dsDNA = 50 μg ml^−1^.

#### AGE

Gels were prepared with 1.5 weight (wt) % agarose in TBE buffer (22.25 mM tris base, 22.25 mM boric acid, 0.5 mM EDTA, and 6 mM magnesium acetate) and cast without stain. Gels were run at 3 V/cm in a water-cooled CBS Scientific HSU-020 gel electrophoresis chamber set to 15°C for 2.5 hours using an Enduro 300 V power source. Gels were imaged once with light-emitting diodes and filters set for Cy3 and Cy5, then poststained in 1× SYBR, and imaged again using an INTAS ECL ChemoStar.

#### DSD simulations

DSD circuits were simulated using the software Visual DSD (*21*) with the programming language reported by Phillips and co-workers (*23*). The codes used are listed in the Appendix. Assuming a standard unbinding rate *u* of 0.012 s^−1^, the binding rates *k* in nM^−1^ s^−1^ are calculated for each reaction. The rate-determining step is the binding to the respective toehold, which is calculated from the melting temperatures at 10 mM Mg^2+^, 5 mM Na^+^, and 160 nM DNA given by the IDT OligoAnalyzer.

#### Fluorescence spectroscopy

For fluorescence intensity measurements, a black 384-well plate from Costar Corning was used. Each well contained 20 μl of solution with 5 mM tris, 1 mM EDTA, 5 mM NaCl, and 10 mM MgCl_2_ at pH 7.2. Evaporation was reduced by adding 4 μl of hexadecane on top.

Fluorescence spectroscopy was done with the Tecan Spark plate reader in top mode. The excitation/emission wavelength was set to 550 nm/570 nm for Cy3 and 645 nm/670 nm for Cy5. Well plates were preincubated for 5 min at 37°C in the plate reader before the input was added. Each measurement was done at least in duplicate, and the average and the SD were calculated (*43*).

#### Transmission electron microscopy

One microliter of sample was diluted with 3 μl of Milli-Q water, incubated for 30 s on plasma-cleaned copper grid, then blotted away using filter paper. For negative staining, 3 μl of 1 wt % uranyl acetate solution was incubated on the grid for 25 s before being blotted away. TEM images were taken with an FEI Talos L120C operating at 120 kV. Nanotubes and fibrils were counted using ImageJ. For each sample, an average of 200 species per origami type were counted manually. The difference in origami dimension and the presence of a cavity enabled easy identification. To prevent confirmation bias, the data were evaluated by two separate researchers. Objects are highlighted for the convenience of the reader in the TEM images, but these highlights do not represent all objects counted. The count was normalized by normalizing the most abundant species at each sample to 100.

#### Folding of DNA origami

The origamis were designed with the program cadnano (*58*), and their design confirmed with the software cando (*32*, *59*). Typical folding mixtures contained 20 nM scaffold, a 5× excess of staple strands (100 nM) and activator/inhibitor strands, and 22 mM MgCl_2_ for cylinders and 5 mM MgCl_2_ for cuboids (*43*).

DNA origamis were folded in a Biometra TPersonal Thermocycler.

Temperature ramp for annealing: 80°C: 15 min, 80° to 60°C: 5 min per 1°C, 60° to 40°C: 3 hours per 1°C, 40° to 25°C: 1 hour per 1°C, stay at 4°C, Lid temp: 80°C.

Folded DNA origamis were stored at 4°C until further use. The folded origami mixtures were purified by spin filtration with Amicon 100-kDa spin filters at 10,000*g* and 15°C for 5 min. The samples were washed six times with FoB5 buffer [5 mM tris, 1 mM EDTA, 5 mM NaCl, and 5 mM MgCl_2_ (pH 7.2)] (*60*) and recovered by turning the filter upside down into a fresh tube and centrifuging at 5000*g* for 3 min.

#### DSD cascades

All DNA sequences for the DSD circuit were diluted from 100 μM stock solutions in TE buffer to 2 to 8 μM in FoB5 buffer and stored at 4°C until further use.

As reference for ssDNA measurements, the unquenched species of c1/input/c1* and c2/activator/c2* were annealed directly at 10 mM MgCl_2_ and used at the respective concentration for normalization of fluorescence measurements over 18 hours.

As reference for origami measurements, input instead of activator was added to the cylinder folding mixture, and activator instead of inhibitor to the cuboid folding mixture before origami folding. These fibrils were diluted to the respective concentration at 10 mM MgCl_2_ and measured as maximum fluorescence compared to DSD reactions run at the same concentration (fig. S5).

For ssDNA measurements, c1/activator (1:1) and c2/inhibitor (1:1) as well as threshold gate were preannealed separately by heating to 95°C and subsequently cooled to 25°C in 20 min, kept at 4°C, and then mixed with the other reactants at 10 mM MgCl_2_. The fluorescence at *t* = 0 min was measured and the reaction started by addition of input.

For origami measurements, cylinders were folded with activator and cuboids were folded with inhibitor and purified. The origamis were then mixed with the other reactants. The fluorescence at *t* = 0 min was measured, and the reaction was started by addition of input. For TEM samples, reactions were prepared identically to fluorescence measurements but incubated in an Eppendorf ThermoMixer C set to 37°C and 300 rpm.

## Supplementary Materials

### This PDF file includes:

Figs. S1 to S8 Tables S1 to S9 Notes S1 to S3

## Notes

View/request a protocol for this paper from *Bio-protocol*.
