# Supplementary material for: Molecular communication relays for dynamic cross-regulation of self-sorting fibrillar self-assemblies
Source: Sci Adv. 2021 Nov 24;7(48):eabj5827. doi: 10.1126/sciadv.abj5827 (PMC8612681; doi:10.1126/sciadv.abj5827)
Supplement: Supplementary file 1 — Figs. S1 to S8 Tables S1 to S9 Notes S1 to S3 [file sciadv.abj5827_sm.pdf]

Science Advances
AAAS

Supplementary Materials for

Molecular communication relays for dynamic cross-regulation of self-sorting fibrillar self-assemblies

Saskia Groeer, Katja Schumann, Sebastian Loescher, Andreas Walther*

*Corresponding author. Email: andreas.walther@uni-mainz.de

Published 24 November 2021, *Sci. Adv.* **7**, eabj5827 (2021)
DOI: 10.1126/sciadv.abj5827

This PDF file includes:

Figs. S1 to S8
Tables S1 to S9
Notes S1 to S3

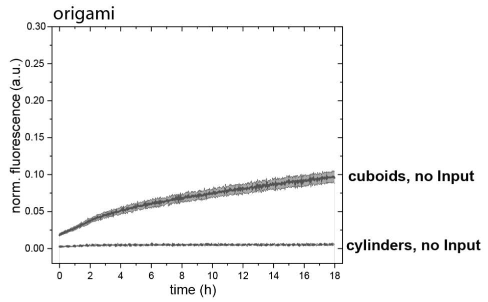

Figure S1. Leakage check without Input addition. Time-resolved normalized fluorescence of 10 nM cuboids (solid line) and 10 nM cylinders (dotted line) without Input addition for reference. All fluorescence measurements are an average of two, the shaded area is the standard deviation.

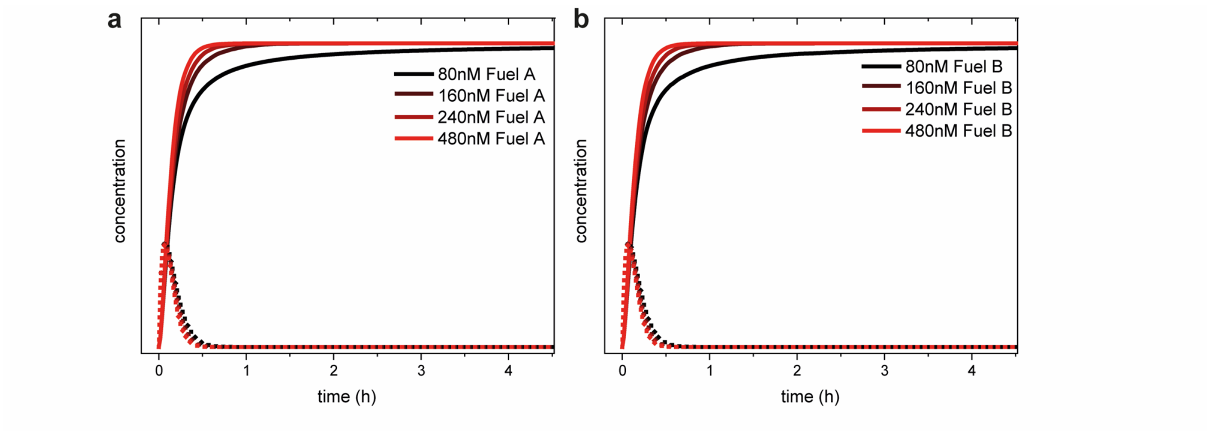

Figure S2. DSD simulations of the amplification module for all fuel concentrations. DSD predicts the behavior of the unquenched Cy3 species (solid lines) and unquenched Cy5 species (dotted lines). c1/Activator, c1*, Input at 160 nM, Inhibitor/c2, c2* at 240 nM. (a) DSD simulations of Fuel A. (b) DSD simulations of Fuel B.

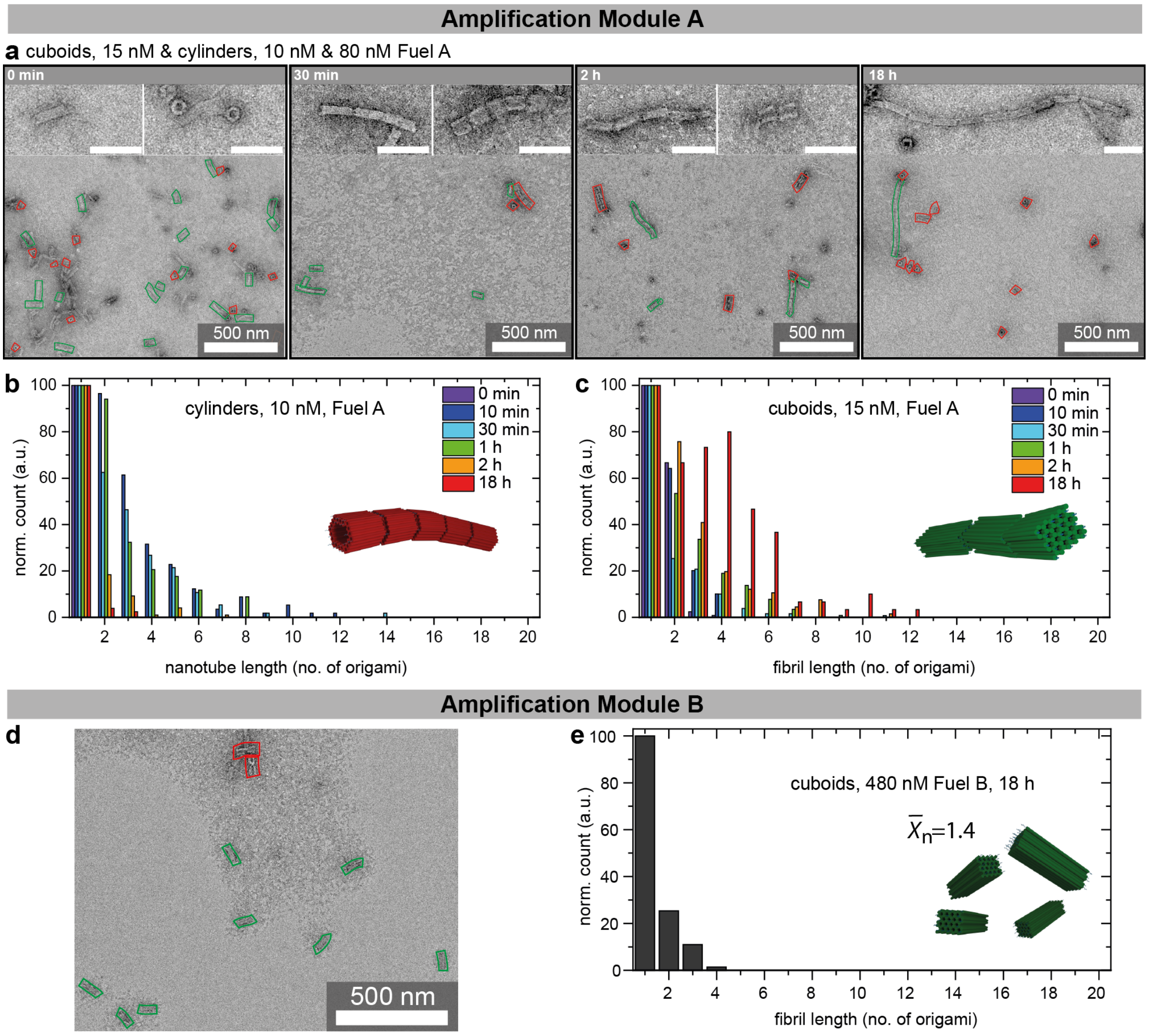

Figure S3. TEM image analysis of the amplification modules. (a) TEM images of DNA origami polymerization at 0 min, 30 min, 2 h and 18 h in presence of 80 nM Fuel A using 15 nM cuboids (green) and 10 nM cylinders (red). Scale bars of insets are 100 nm. **(b)** Statistical TEM image analysis of nanotube lengths in presence of 80 nM Fuel A using 15 nM cuboids and 10 nM cylinders. **(c)** Statistical TEM image analysis of cuboid fibril lengths in presence of 80 nM Fuel A using 15 nM cuboids and 10 nM cylinders. **(d)** TEM image of DNA origami polymerization after 18 h in presence of 480 nM Fuel B using 15 nM cuboids (green) and 10 nM cylinders (red). **(e)** Statistical TEM image analysis of cuboid fibril lengths after 18 h with 480 nM Fuel B. Colored lines are added as guide to the eye for identifying both origami types. Scale bar of inset is 100 nm.

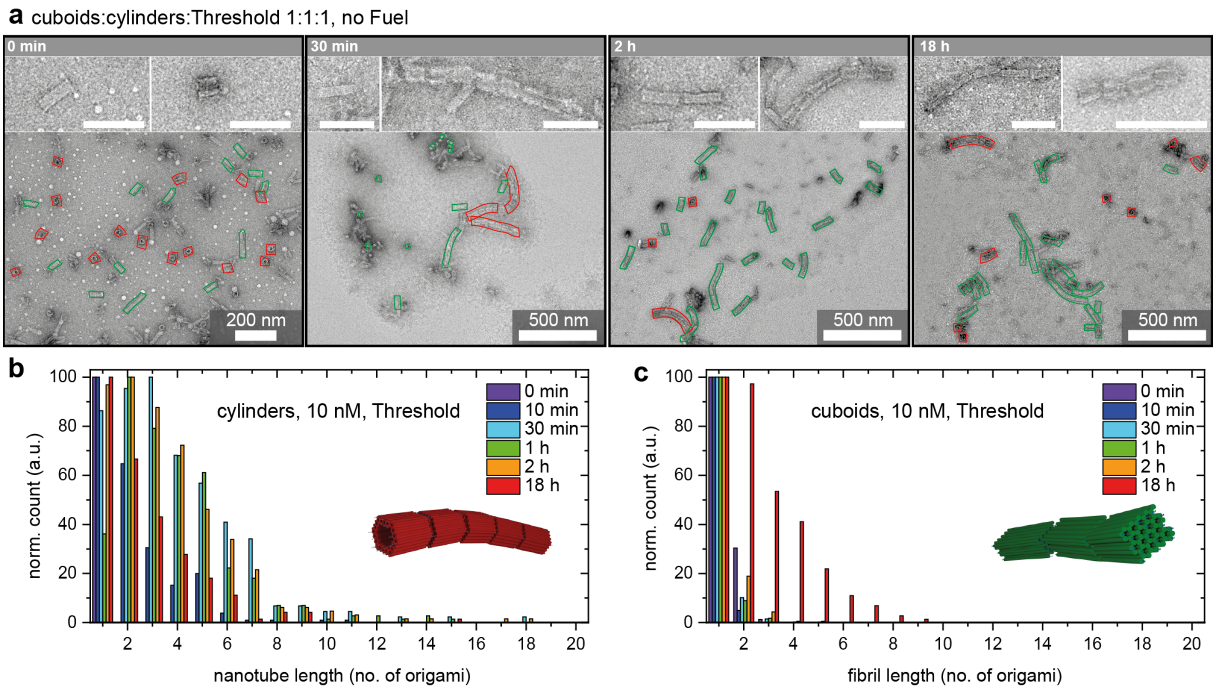

Figure S4. TEM image analysis of the threshold module. (a) TEM images of DNA origami polymerization at 0 min, 30 min, 2 h and 18 h in presence of 160 nM Threshold using 10 nM cuboids (green) and 10 nM cylinders (red). Colored lines are added as guide to the eye for identifying both origami types. Scale bars of insets are 100 nm. **(b)** Statistical TEM image analysis of nanotube length distribution in presence of 160 nM Threshold using 10 nM cuboids and 10 nM cylinders. **(c)** Statistical TEM image analysis of cuboid fibril length distribution in presence of 160 nM Threshold using 10 nM cuboids and 10 nM cylinders.

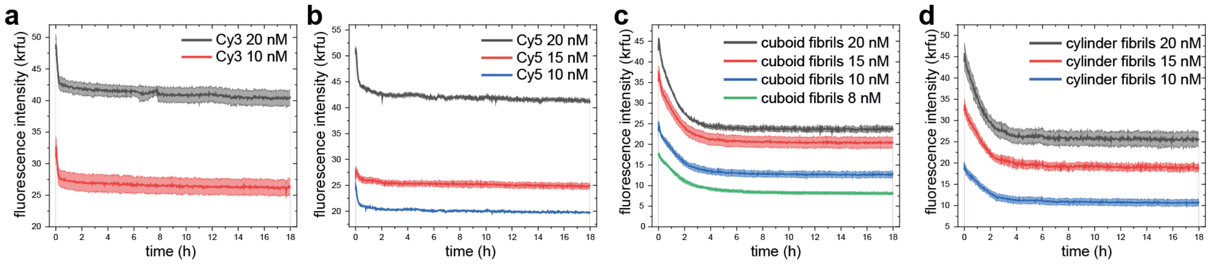

Figure S5. Reference measurements for normalizing fluorescence. (a) Time-resolved fluorescence of the c2(Cy3)/Activator/c2* complex. **(b)** Time-resolved fluorescence of the c1(Cy5)/Input/c1* complex. **(c)** Time-resolved fluorescence of cuboid fibrils pre-formed during origami folding and purified for normalizing. **(d)** Time-resolved fluorescence of cylinder nanotubes pre-formed during origami folding and purified for normalizing.

Note S1. Visual DSD code for the negative feedback loop.

```
directive simulation {
 final=65000;
}
directive simulator deterministic

directive rules {
bind(P1,P2,Q,D!i) :-
P1 = C1 [D@X], compl(D, D'), P2 = C2 [D'@Y],
Q = C1 [D!i] | C2 [D'!i], freshBond(D!i, P1|P2),
not hidden(D@X, P1),
not hidden(D'@Y, P2).
bind(P,Q,D!i) :-
P = C [D @ X][D' @ Y], compl(D, D'),
Q = C [D!i] [D'!i], freshBond(D!i, P),
not hidden(D @ X, P),
not hidden(D' @ Y, P).
displace(P,Q,E!j,D!i) :-
P = C [E!j D] [D!i] [D'!i E'!k], junction(E!j, E'!k, P),
Q = C [E!j D!i] [D] [D'!i E'!k].
displaceL(P,Q,E!j,D!i) :-
P = C [D!i] [D E!j] [E'!k D'!i], junction(E!j, E'!k, P),
Q = C [D] [D!i E!j] [E'!k D'!i].
unbind(P,Q,D!i) :-
P = C [D!i] [D'!i], toehold(D),
Q = C [D] [D'], not adjacent(D!i,_,P).
adjacent(D!i,E!j,P) :- P = C [D!i E!j] [E'!j D'!i].
adjacent(D!i,E!j,P) :- P = C [E!j D!i] [D'!i E'!j].
```

```
cover(P,Q,E!j,D!i) :-
P = C [E!j D] [D' E'!j], compl(D, D'),
Q = C [E!j D!i] [D'!i E'!j], freshBond(D!i,P).
coverL(P,Q,E!j,D!i) :-
P = C [D E!j] [E'!j D'], compl(D, D'),
Q = C [D!i E!j] [E'!j D'!i], freshBond(D!i,P).
binds(P1,P2,R,D!i,[D#L]) :- bind(P1,P2,Q,D!i), not coverL(Q,_,D!i,_), covers(Q,R,D!i,L).
binds(P1,P2,Q,D!i,[D]) :- bind(P1,P2,Q,D!i), not coverL(Q,_,D!i,_), not cover(Q,_,D!i,_).
displaces(P,R,E!j,[D#L]) :- displace(P,Q,E!j,D!i), displaces(Q,R,D!i,L).
displaces(P,Q,E!j,[D]) :- displace(P,Q,E!j,D!i), not displace(Q,_,D!i,_).
displacesL(P,R,E!j,[D#L]) :- displaceL(P,Q,E!j,D!i), displacesL(Q,R,D!i,L).
displacesL(P,Q,E!j,[D]) :- displaceL(P,Q,E!j,D!i), not displaceL(Q,_,D!i,_).
covers(P,R,E!j,[D#L]) :- cover(P,Q,E!j,D!i), covers(Q,R,D!i,L).
covers(P,Q,E!j,[D]) :- cover(P,Q,E!j,D!i), not cover(Q,_,D!i,_).
coversL(P,R,E!j,[D#L]) :- coverL(P,Q,E!j,D!i), coversL(Q,R,D!i,L).
coversL(P,Q,E!j,[D]) :- coverL(P,Q,E!j,D!i), not coverL(Q,_,D!i,_).
unbinds(P,R,D!i,[D#L]) :-
P = C [D!i E!j] [E'!j D'!i], toehold(D), not boundL(D!i,_,P),
Q = C [D E!j] [E'!j D'], unbinds(Q,R,E!j,L).
boundL(D!i,E!j,P) :- P = C [E!j D!i] [D'!i E'!j].
unbinds(P,Q,D!i,[D]) :- unbind(P,Q,D!i).
(* auxiliary functions *)
hidden(D@X, P) :-
unbound(D),
P = C [A@End D@X B@Start],
path(B@Start, _@End, P, "right", [], Path).
hidden(D@X, P) :-
unbound(D),
P = C [B@Start D@X A@End],
path(B@Start, _@End, P, "left", [], Path).
(* path predicate: find a @Path from @Start to @End *)
path(_@End, _@End, _, _, Visited, Path) :-
reverse(Visited, Path).
path(X@Start, _@End, P, "left", Visited, Path) :-
not (Start = End),
P = C[Y@Start' X@Start],
not member(Y@Start', Visited),
path(Y@Start', _@End, P, "left", [X@Start # Visited], Path).
path(X@Start, _@End, P, "right", Visited, Path) :-
not (Start = End),
P = C[X@Start Y@Start'],
not member(Y@ Start', Visited),
path(Y@Start', _@End, P, "right", [X@Start # Visited], Path).
path(X@Start, _@End, P, "any", Visited, Path) :-
not (Start = End),
path(X@Start, _@End, P, "left", Visited, Path).
path(X@Start, _@End, P, "any", Visited, Path) :-
not (Start = End),
path(X@Start, _@End, P, "right", Visited, Path).
path(D!i@Start, _@End, P, _, Visited, Path) :-
not (Start = End),
P = C [D!i@Start] [D'!i@Start'],
not member(D'!i@Start', Visited),
path(D'!i@Start', _@End, P, "any", [D!i@Start # Visited], Path).
junction(A, B, P) :- junctionR(A, B, P, []).
junction(A, B, P) :- junctionL(A, B, P, []).
```

```
junctionR(_!j,_!j,_,_).
junctionR(E!j,F!k,Q,V):-
Q = C [F!k] [G!l E'!j] [E!j],
not member(X, V),
not member(Y, V),
junctionR(G!l,F!k,Q,[X;Y#V]).
junctionL(_!j,_!j,_, _).
junctionL(E!j,F!k,Q, V):-
Q = C [F!k] [E'!j@X G!l@Y] [E!j],
not member(X, V),
not member(Y, V),
junctionL(G!l,F!k,Q,[X;Y#V]).
(* infinite semantics *)
find(D,Type,Rate):- rate(D,Type,Rate).
find(D,Type,Rate):- default(D,Type,Rate), not rate(D,Type,_).
//order of find and binds clauses could be reversed.
slow(P1, P2, Rate, Q) :- find(L, "bind", Rate), binds(P1,P2,Q,_,L). //, productive(Q,_,_!i,L).
fast(P, Rate, Q) :- displaces(P,Q,_,L), find(L, "displace", Rate).
fast(P, Rate, Q) :- displacesL(P,Q,_,L), find(L, "displace", Rate).
fast(P, Rate, Q) :- covers(P,Q,_,L), find(L, "cover", Rate).
fast(P, Rate, Q) :- coversL(P,Q,_,L), find(L, "cover", Rate).
fast(P, Rate, Q) :- unbinds(P,Q,_,L), find(L, "unbind", Rate).
productive(P,Q,E!j,L) :- displaces(P,Q,E!j,L).
productive(P,Q,E!j,L) :- displacesL(P,Q,E!j,L).
productive(P,Q,E!j,L) :- covers(P,Q,E!j,L).
productive(P,Q,E!j,L) :- coversL(P,Q,E!j,L).
mergestep(P,Q,V) :- fast(P,_,Q), not member(Q,V).
merge(P,P,V) :- not fast(P,_,_).
merge(P,R,V) :- mergestep(P,Q,V), merge(Q,R,[Q#V]).
reaction([P1; P2], Rate, R) :- slow(P1, P2, Rate, Q), merge(Q,R,[(P1|P2);Q]).
reaction([P], Rate, R) :- slow(P, Rate, Q), merge(Q,R,[P;Q]).
infinite([P1; P2], Rate, R) :- slow(P1, P2, Rate, Q), merge(Q,R,[(P1|P2);Q]).
infinite([P], Rate, R) :- slow(P, Rate, Q), merge(Q,R,[P;Q]).
detailed([P1; P2], Rate, Q):- slow(P1, P2, Rate, Q).
detailed([P], Rate, Q) :- slow(P, Rate, Q).
detailed([P], Rate, Q) :- fast(P, Rate, Q).
default([_],"unbind",0.012).
default(_,"bind",0.00002).
default(_,"displace",1.0).
default(_,"cover",1.0).
}

( 160 [<a^!1 b^!2 c^!3 d^!4 e^>
   | <h* g^* d^*!4 c^*!3 f^* b^*!2 a^*!1>]
| 160 [<e^* d^* c^* b^* i*>]
| 160 [<i>]
| 160 [<f^ c^!11 d^!12 g^!13 k^!14>
   |<k^*!14 g^*!13 e^* d^*!12 c^*!11 b^* a^*>]
| 160 [<h>]
)
```

Note S2. CRN code for the basic negative feedback loop.

```
directive simulation {final=65000; plots=[sp_0; sp_1; sp_2; sp_3; sp_4; sp_5;
sp_6; sp_7; sp_8; sp_9; sp_10; sp_11; sp_12; sp_13; sp_14]; }
directive simulator deterministic

| 160 sp_0
| 160 sp_1
| 160 sp_2
| 160 sp_3
| 160 sp_4
| sp_1 + sp_2 ->{0.00015} sp_14
| sp_0 + sp_4 ->{0.00013} sp_11
| sp_1 + sp_0 ->{0.000041} sp_13 + sp_12
| sp_14 + sp_0 ->{0.000041} sp_10 + sp_12
| sp_13 + sp_2 ->{0.00015} sp_10
| sp_12 + sp_4 ->{0.00013} sp_7
| sp_3 + sp_12 ->{0.000021} sp_9 + sp_8
| sp_1 + sp_11 ->{0.000041} sp_13 + sp_7
| sp_14 + sp_11 ->{0.000041} sp_10 + sp_7
| sp_9 + sp_4 ->{0.00013} sp_6
| sp_0 + sp_8 ->{0.000041} sp_12 + sp_5
| sp_13 + sp_8 ->{0.000062} sp_1 + sp_5
| sp_8 + sp_11 ->{0.000041} sp_5 + sp_7
| sp_10 + sp_8 ->{0.000062} sp_14 + sp_5
| sp_3 + sp_7 ->{0.000021} sp_8 + sp_6
```

Note S3. CRN code for the negative feedback loop in presence of Fuel A.

```
directive simulation {final=65000; plots=[sp_0; sp_1; sp_2; sp_3; sp_4; sp_5;
sp_6; sp_7; sp_8; sp_9; sp_10; sp_11; sp_12; sp_13; sp_14; sp_15; sp_16; sp_17;
sp_18]; }
directive simulator deterministic

| 160 sp_0
| 160 sp_1
| 160 sp_2
| 240 sp_3
| 240 sp_4
| 240 sp_5
| sp_4 + sp_5 ->{0.00013} sp_18
| sp_1 + sp_2 ->{0.00014} sp_17
| sp_0 + sp_4 ->{0.00013} sp_14
| sp_1 + sp_0 ->{0.000041} sp_16 + sp_15
| sp_17 + sp_0 ->{0.000041} sp_13 + sp_15
| sp_16 + sp_2 ->{0.00014} sp_13
| sp_15 + sp_4 ->{0.00013} sp_10
| sp_3 + sp_15 ->{0.000021} sp_12 + sp_11
| sp_1 + sp_14 ->{0.000041} sp_16 + sp_10
| sp_17 + sp_14 ->{0.000041} sp_13 + sp_10
| sp_12 + sp_4 ->{0.00013} sp_8
| sp_0 + sp_11 ->{0.000041} sp_15 + sp_6
| sp_16 + sp_11 ->{0.000062} sp_1 + sp_6
| sp_11 + sp_14 ->{0.000041} sp_6 + sp_10
| sp_13 + sp_11 ->{0.000062} sp_17 + sp_6
| sp_3 + sp_10 ->{0.000021} sp_11 + sp_8
| sp_12 + sp_18 <->{0.000040}{0.000021} sp_9 + sp_15
| sp_9 + sp_10 <->{0.000021}{0.000041} sp_18 + sp_8
| sp_7 + sp_4 ->{0.00013} sp_9
| sp_12 + sp_5 <->{0.00004}{0.000021} sp_7 + sp_15
| sp_5 + sp_8 <->{0.00004}{0.000021} sp_7 + sp_10
```

Note S4. CRN code for the negative feedback loop in presence of Threshold.

```
directive simulation {final=65000; plots=[sp_0; sp_1; sp_2; sp_3; sp_4; sp_5;
sp_6; sp_7; sp_8; sp_9; sp_10; sp_11; sp_12; sp_13; sp_14; sp_15; sp_16; sp_17;
sp_18; sp_19; sp_20; sp_21; sp_22]; }
directive simulator deterministic

| 160 sp_0
| 160 sp_1
| 160 sp_2
| 160 sp_3
| 160 sp_4
| 160 sp_5
| 160 sp_6
| sp_4 + sp_5 ->{0.00013} sp_22
| sp_1 + sp_2 ->{0.00014} sp_21
| sp_0 + sp_4 ->{0.00013} sp_18
| sp_1 + sp_0 ->{0.000041} sp_20 + sp_19
| sp_21 + sp_0 ->{0.000041} sp_17 + sp_19
| sp_20 + sp_2 ->{0.00014} sp_17
| sp_6 + sp_19 ->{0.000039} sp_12 + sp_19
| sp_6 + sp_19 ->{0.000039} sp_13
| sp_19 + sp_4 ->{0.00013} sp_14
| sp_3 + sp_19 ->{0.000021} sp_16 + sp_15
| sp_1 + sp_18 ->{0.000041} sp_20 + sp_14
| sp_21 + sp_18 ->{0.000412} sp_17 + sp_14
| sp_16 + sp_4 ->{0.00013} sp_10
| sp_0 + sp_15 ->{0.000041} sp_19 + sp_8
| sp_20 + sp_15 ->{0.000062} sp_1 + sp_8
| sp_15 + sp_18 ->{0.000041} sp_8 + sp_14
| sp_17 + sp_15 ->{0.000062} sp_21 + sp_8
| sp_6 + sp_14 ->{0.000039} sp_12 + sp_14
| sp_6 + sp_14 ->{0.000039} sp_7
| sp_3 + sp_14 ->{0.000021} sp_15 + sp_10
| sp_13 + sp_4 ->{0.00013} sp_7
| sp_12 + sp_19 ->{0.000039} sp_13
| sp_12 + sp_14 ->{0.000039} sp_7
| sp_16 + sp_22 <->{0.00004}{0.000021} sp_11 + sp_19
| sp_11 + sp_14 <->{0.000021}{0.00004} sp_22 + sp_10
| sp_9 + sp_4 ->{0.00013} sp_11
| sp_16 + sp_5 <->{0.00004}{0.000021} sp_9 + sp_19
| sp_5 + sp_10 <->{0.00004}{0.000021} sp_9 + sp_14
```

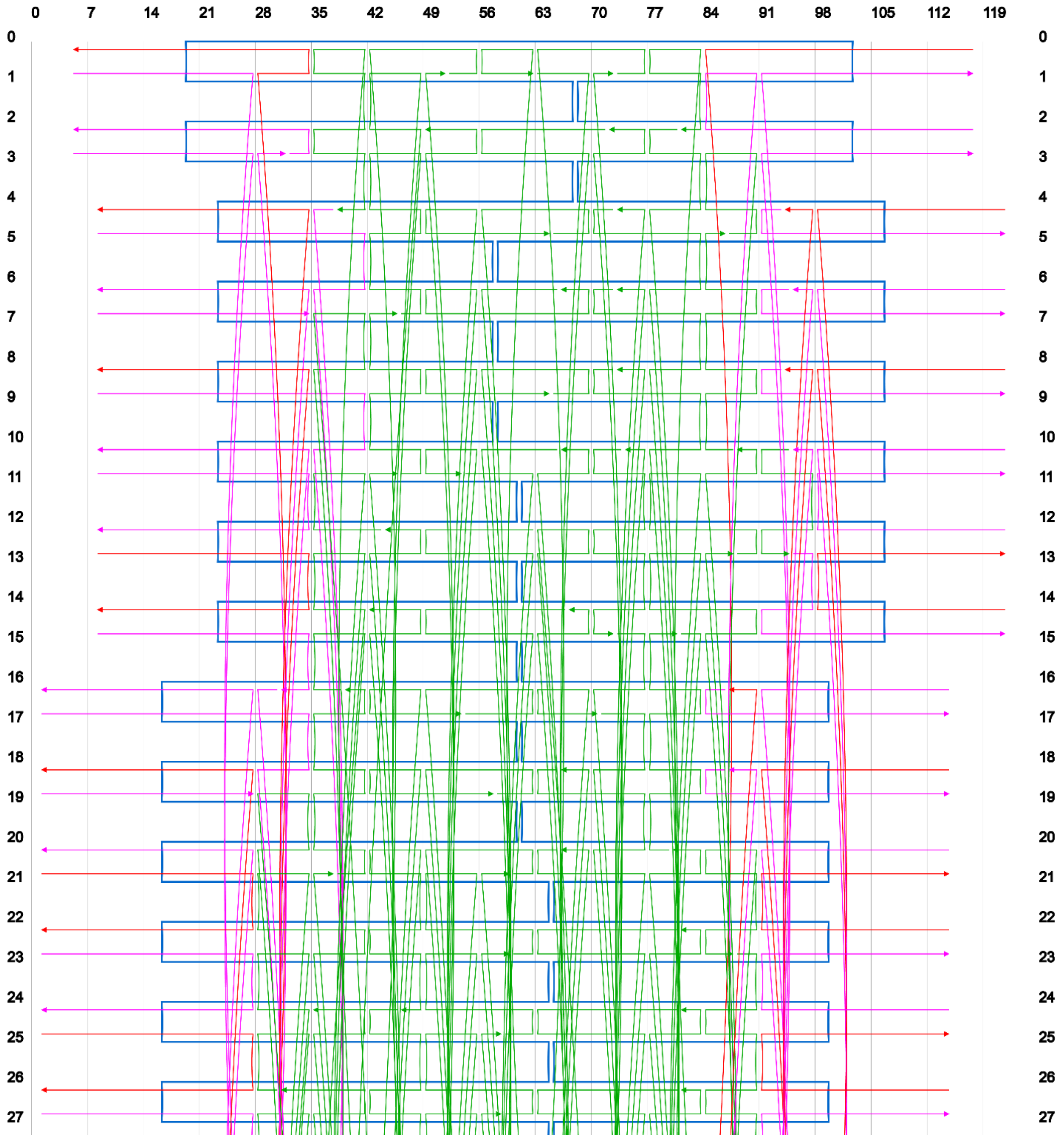
0 7 14 21 28 35 42 49 56 63 70 77 84 91 98 105 112 119
0 1 2 3 4 5 6 7 8 9 10 11 12 13 14 15 16 17 18 19 20 21 22 23 24 25 26 27

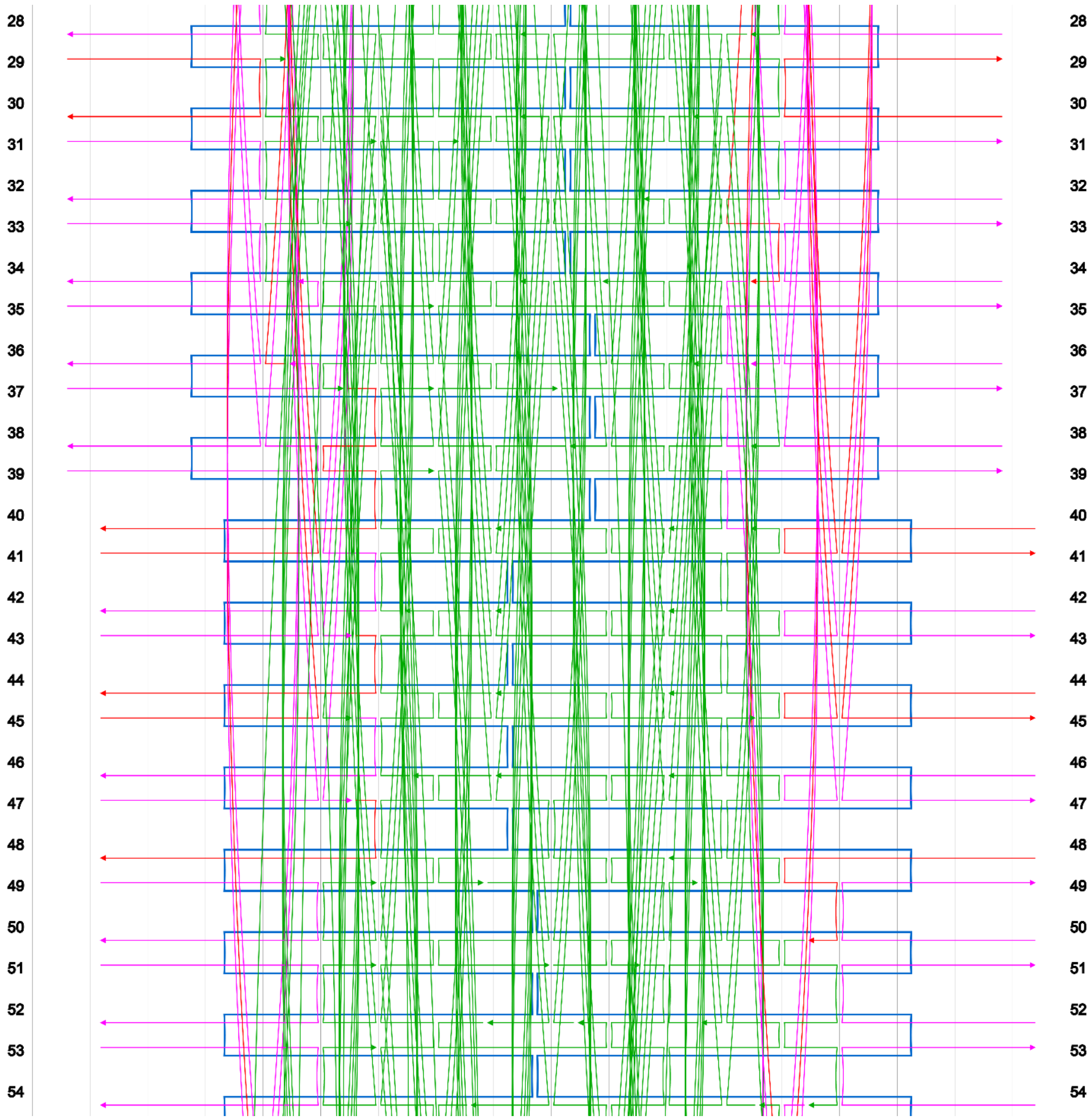
28
29
30
31
32
33
34
35
36
37
38
39
40
41
42
43
44
45
46
47
48
49
50
51
52
53
54

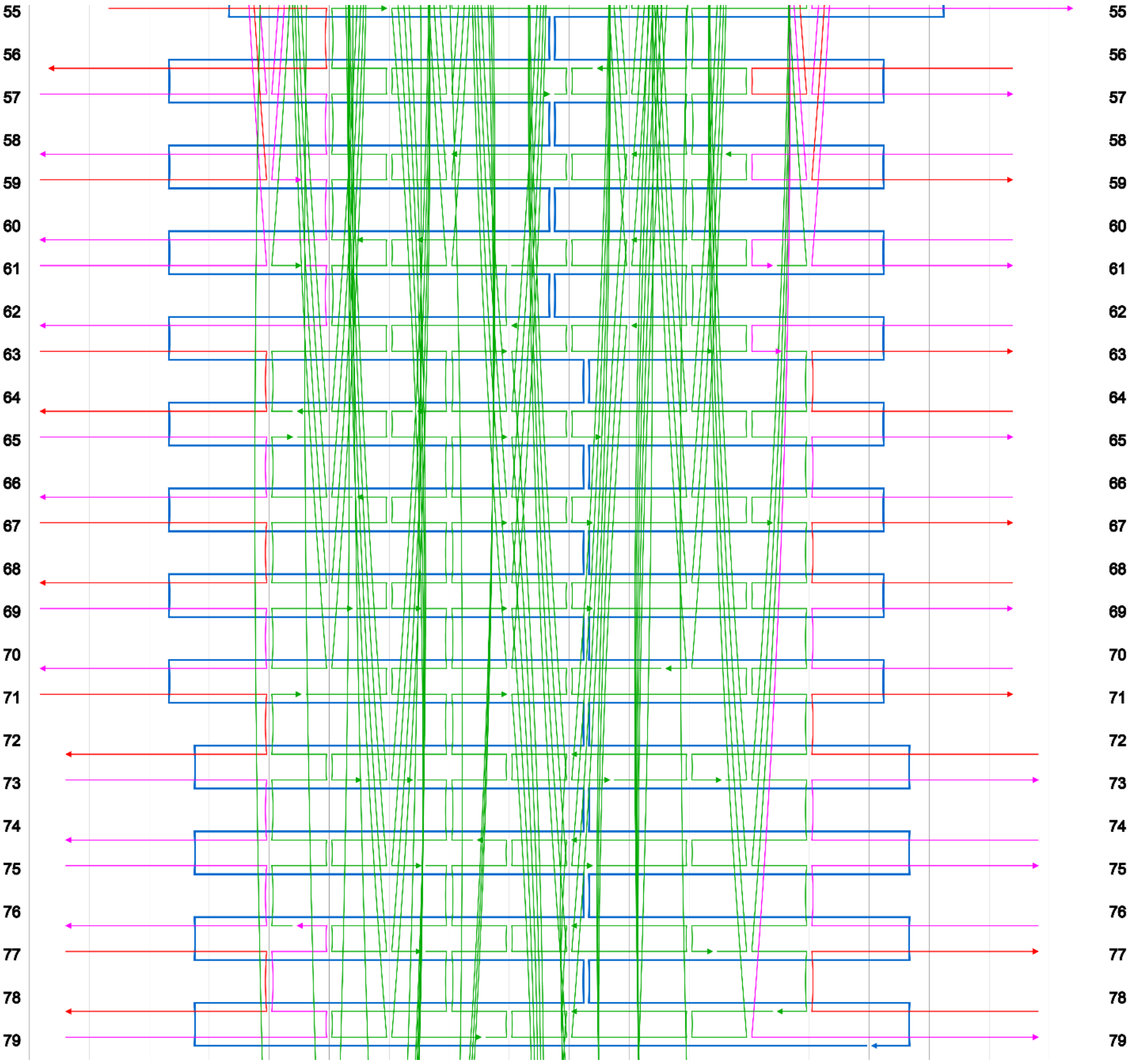

Figure S6. 3D DNA origami cylinder folding map. Scaffold is depicted in blue, core staple strands in green, poly-thymine passivated strands in pink and connector overhangs in red (16 connectors per side).

Table S1. Sequences of core staples in cylinders. Names denote both the function of the staple strand and its position according to the line numbers in Figure S5: [function- 5' position – 3' position].

Name	Core staples 5'→3'
**core-2-42**	GTG CGG AGT GTG TCA TAC AAA AGG TCA GAG GC
**core-69-73**	CCC ATA GCT GGA AGC ATG CTA ACT CAC ATT AGG GA
**core-61-60**	ATT CTC CGA GAA CGC CAT CAA AAA T
**core-29-69**	TAT TTT AAT AAA TTA CCT TGT GAA AAG GTC GAC TC
**core-65-21**	GGA AAC CAG GCG ATC GCA TCC
**core-26-66**	GTT CTG CTC AAC GAC GGA GGG GGA TGT GCT GTC GG
**core-77-37**	TGA AAT CAA AAG AAT AGG AAC AAG ATT CAT TAA ACG AGC AAA GCG AAG
**core-20-65**	TAC GGT AAG AAA AGT AAC GAC AAC TCG TAG AGC GA

**core-58-19**	ATT TAA ACT CAT TTT GCC ACT ATC TTT GAC CCC CAG C
**core-31-69**	CCC TAC ATT CAT CAT CAG CGT TGG GAA CTG GCC ATG GTC CGG GTA C
**core-69-24**	TAG ATT GTA AAT TCA GTG AGA ACC G
**core-42-39**	GAT GTC TGG AAA TGC TGA AGA GGT
**core-5-38**	CAG AAG TAA GCA CTG GTA CTA A
**core-42-6**	ATA TAA CTT TCG CAC ATA GTT TCT GTA TGG TCA GAG CCA CCA TTG G
**core-54-52**	AGC ATC AAC CGT TAT TCA TTA AAG GGG GAA TTA AT
**core-76-71**	GGT TCT TTT CGC GTA TTT CCA GTC ATT GCG TCA AC
**core-21-16**	GCG AAG TAC AAA ACA CTC ACG AAG GTC ATG AGC GA
**core-61-23**	TTC CCC GAG GAG CCG AAC AAT AGC TTA AG
**core-37-1**	GCT TCA ATC AAT AAC TCA TCG TT
**core-56-17**	ATT TAG ATT TGT TTA CCT ATA AAA G
**core-60-55**	GTC AAA TCA GTT GTA AAA GAA AAG ATC GTA ACA TTG CC
**core-46-11**	GTA GTA GCA ATA AAA ATA ATT TAT CG
**core-32-1**	GAT ATA GCG TGA GAA GGG AAT AGG
**core-1-32**	AAT GCT GAG ACT CCT CAA CCA GGC TTA TCC GGT GA
**core-2-38**	TAA ACA CCG TAC TCA GGG GAT AGC AAG CGA ACC AA
**core-17-57**	AAA CGG GCA AGG CGA AAG ACA GCA ACA GGA AGA TT
**core-73-72**	AGT CTT TAA CCA GCA GAA GAT GG
**core-30-75**	ACC CGT TTA CCA TCA AGA TTA GTT GCG TCT TTC CAG AGC CT
**core-52-10**	CAA TCG TCG CTA TTA ATT ATA GCT TCG ACA GAC GT
**core-70-26**	CAG CAA ATG ATG GAA AAC AGA GGT GAG GCG GTT GAG AGC AAA TAG CGA A
**core-15-13**	CAT AAA GAA GAT TAC ATT TAA CAA GTA CAT AAG AG
**core-71-26**	ATA CGA GCC GTT TCC TGT ATG CGA GGC TTG AGA TG
**core-4-44**	CTT TAA ATC CTC ATT AAG GCA GGT GTA GGG CTA TGC GT
**core-63-67**	TTA CTT TAC ACA TTT GAT AGG AGC TGA
**core-49-45**	CAT AGG TCT GGG GTT ATG AAA ACT TGA CCT AAT AA
**core-1-79**	CCC GAT TAG GAT TAG CGG AAT GTT TCA TAA ATA GTC CAC TAT T
**core-78-36**	GCT CAT GGA AAG GGC GAC AAG CAA AGC CGT TGT AC
**core-66-64**	TGC TGT ACA GTA AGG GAA CCG AAC GTC GAA ACT CC
**core-25-65**	CGA GTC ATC AAG AGT AAT GCA TAG GGT TGG GAC ATT CGC
**core-67-73**	GGA ATC AAT CAT TGC TGA GCC ACG CCA GTA TTT ACC GAA CGA ACC ATG CG
**core-18-62**	GGC AAG AAC TGG CAT GAT AAT AAC GCG GAA TCG GAA CA
**core-14-54**	CCG TCA CGC ATA ACC GAT TCA CCC TCA GCA CGA TAA TGC CGG
**core-49-46**	GCC TTT AAT AGT GAA ATG CTG AAT CGC AAA AAT ACC GAC CGT A
**core-33-4**	TAA GGT TTT GGG TTG ATC CAC CCT GAG CCA CCT GA
**core-6-42**	CCT TGA TAC TAA ATA AAG CCA ACG AGC CAG TAA TAA CC

**core-34-78**	TAG AAA GAC TCC AAC GTC AAA TAC CTA CAT TTG G
**core-60-24**	AAT TAA CAA CGA AAC AAC CTG CTC TCA ATC AAC CAG GCC TT
**core-19-58**	GAT AAA ATA AAA TAC GTA ATT TAA CCA ATA GTT TGG TAT AAG CAA AT
**core-38-2**	AGC GAA CCA GCG GAT GGC TTA TTT ATC AAC TGT AGA ATG ATA CAC CGT A
**core-20-15**	CAA TTA AGA CAA ATA CAA AAG ACA AGA AAA TGC GA
**core-44-9**	GTC AAT ATT CTA CTT TTT GCT GAA TTG CGA TAT TAG C
**core-45-40**	ACA AGT ATC ATT AAT TGA TTT AGG AAA GTA ATC AG
**core-23-28**	AGC TAA TAT CAC AAA GTA AAC AGG AGC CTT TCA AA
**core-5-40**	ACA AAT TCC AAT AGG AAC CTT GAG AAT ATA AAG TGG AGC TTA ATT GC
**core-24-69**	GAG ATA ACC CAC AAG ACT AAA ATT AAC TGA ACG TCA GTT GGC AAC CGA A
**core-36-40**	CGC ATC GGC TAC GAG CAA ATA GAT ATG CAG A
**core-64-69**	GAA AAA GCG CAG GGC GAC AAG GCG CAC GAC GGG AT
**core-27-67**	TTA TAC CAG AAC GAG TAG CTT GCC CTC CCA GTA TTA AGT
**core-50-54**	GCG AAT TTT CGG AAA CAT TTC ATT CAA T
**core-21-64**	GCC CCT ATA CCA AGC GCC CGT CGG GGA ACA AAC GGC GGT GCA TCT TCA G
**core-75-74**	CTG GCC AAC AGT TTT GAA TGG CTA A
**core-73-75**	AAC ATC TAC GAC AGG TAG AAA GAT ACT AAT GAG AC
**core-65-20**	GCC GTC AAT AGA TAA TAA ACA ATT GCA GAT AAA CG
**core-10-13**	AGC GAT CAA GTC ATT AGC GCA A
**core-67-22**	TCA ACA GTT GAA AGG AAT ACT AAC ACC AAT AAA TC
**core-9-44**	GTT TGG AAC CGC CAC CCG AAA TGT GAT AAA TAA ATT CTT ACC AGT TG
**core-28-73**	GGA TTG AGA TTT AGC CTA ATT TGC TTT TTT GTT TAA CAT GAT T
**core-6-46**	CGA CCC TCA GAG CCG CCA GCC GCC AAT AAG AAA TTT AA
**core-17-60**	AAA CGC TAC ATA ATG AAT AAA TCC TGA
**core-13-49**	CCA AAG GCC GGA AAC GTT CAG TAG AGA TTA AAA AT
**core-63-58**	GAA CCA TTT TGT ATC ATC CAA TAT ATG GAA GGG TA
**core-51-56**	AAT ATA TGT GAA AAT TAA TGA TGA TTT GAA TAA CAA TAA CGG
**core-30-78**	CGA GCT ATT TTG CAC CCC TTG CGG ATG GAT TAA AC
**core-32-76**	AGC CTT AAA GAT CTG ACG CTC AAT CTA AAA GGG ACA TTT
**core-51-10**	TTG CTT CAG AAG AGT AGC AGC TCA T
**core-7-37**	TTT AGC GTA ACA TTC CAC GTA ACA CAC CCT CAG CAA ACC AAA TAT
**core-16-53**	GGG TCT TTT GCA TTT TTG TAT TCA A
**core-24-73**	GAC AAA TAA GGT AAA TTG TTT TAA GAA GAA AAC TG
**core-12-49**	AAT TTC TTA GGA GCC TAA TTT TTG TAA TAC
**core-10-49**	AGC CCC CTA TGC ATC CAT GCA ACT CAG AGC AGG AGA A
**core-11-51**	GTT TAT CGA TCA TTT CAA CGC AAG TGT AGG TAA AGC C

**core-40-5**	TGA ATA TAG TTT CAC ATG TAC CAG ACA GCC TTC ACA A
**core-79-74**	AAA CCT TAT ATG GTG GTT GGC CCT CAA CAG CAC GC
**core-26-70**	GCG CAT TAG ACG GGA GAC ACC GTC AAA AAT GAC AG
**core-57-17**	CGC GAG AGA ATC GAT GAA CGG TAC CCC AAA ATC GGA AGA AGT TTC CAT T
**core-77-2**	TGA AGA GGT TTT ATT CTA TAA GAG GCC CCC TGA CA
**core-1-37**	TGT ATC AGA TTT ACC CTG ACT TAA TTC GA
**core-28-30**	TAA ACT GAT AGG CAC AGA CAA TAT AGA TAG ACT AA
**core-69-24**	AAA ATC TAA AGC ATC ACC ATA TCT GAC CCT GAA GA
**core-73-31**	TCG TGG GAG AGG CGG TTT ACC AGT GCA GAT ACG AGC AAC
**core-29-77**	AAA ATA AAC CAA CGA CCC TTC CAC ACG ACC AGT AAG TC
**core-10-5**	TTT CTC AAA ATC CCT CAG ACC AGA AAG GTT GAA GC
**core-11-7**	TTG TTT TCA CAC TAA AGA AAC AAC TGA A
**core-75-33**	GGG GAG AGA GTT GCA GCA AAA TCC CGA GAG GAT AG
**core-22-67**	TTA CCG AAG CCC TTT TAA ACA TTG AGT TAA GCA CTA ATA GAT TAT GCG A
**core-12-52**	TCA GCT TGC TTT CGA ACC ATC GCC CAC CAA ATT CAA
**core-34-34**	GCG AAA AAT CAG GTC GAA CAA GCA ATC AGA TA
**core-15-58**	AAG GTC ATA TGT CAG GTT AGA AAT A
**core-8-48**	TCA CCA TCT TTT CAT AAA TCG GCA AAC GCG AAT AAC TA
**core-52-52**	AAG GGT AGC TGA TAA ATT CAA GAA AAC AGT G
**core-23-63**	CTG CGA GGC GCA GAC GGC ATG TTA ATC GGC CGC CAG TT
**core-10-15**	TCG GAC CGT AAC ACC AAT GCC ATT TTG AAT TAC AA
**core-46-42**	TAA CGC ATC AAA CCT GTT AGA TAC AAG TT
**core-64-60**	AGC GCG CAT CGT AAC CGA TTG ACC GAG CGA GTC GC
**core-54-14**	AGA GCT GGA GCA AAC ACC TGA TTG CAA CAA ACC CAA AGA TCA
**core-34-29**	TCC CGG GGG TAC TTT TGC ATA GTA AAT AAC GCC AT
**core-74-71**	GCG GCC AGC TTG AGT GAA AAG TGT
**core-58-54**	AAA CTA ACG TCC GGG AGA ACC AAG TTA CAA AAC TG
**core-100-23**	GAT TGT GAT TGT GAT TGT TTT TTT TTT TGG GTA ACG CAA CTC TGG
**core-87-34**	GAT TGT GAT TGT GAT TGT TTT TTT TTT CAA AAT CGA ACG TGT ACT
**core-101-8**	GAT TGT GAT TGT GAT TGT TTT TTT TTT TAT ACA AGG CGT TAA CCC
**core-86-2**	GAT TGT GAT TGT GAT TGT TTT TTT TTT CAT TTT TGA CCG GAA TTT TCA GAG GTT T
**core-94-14**	GAT TGT GAT TGT GAT TGT TTT TTT TTT TGA GAG TGG TAG CTG GGA TCG ATA TTC G
**core-81-28**	GAT TGT GAT TGT GAT TGT TTT TTT TTT TGC CTA AGC ATT AAG TCA
**core-88-46**	GAT TGT GAT TGT GAT TGT TTT TTT TTT TTT TGC GTA AAG CTA ATT AAG CAT
**core-95-63**	GAT TGT GAT TGT GAT TGT TTT TTT TTT AAG AAA CAC TTA AAT CCT TTG CCC
**core-83-30**	ATT ACT GCA AGT GCA AAT TTT TTT TTT TCG GCC ATG ATT GCG AAT

**core-98-25**	ATT ACT GCA AGT GCA AAT TTT TTT TTT CGA GCT CAG GGT TTT GA
**core-90-11**	ATT ACT GCA AGT GCA AAT TTT TTT TTT GAG TAA TGG ATA AAT TAA
**core-103-10**	ATT ACT GCA AGT GCA AAT TTT TTT TTT TGG TTT GGA CAA AGT TT
**core-97-4**	ATT ACT GCA AGT GCA AAT TTT TTT TTT ACG CGC CTA CCG ACA TGG
**core-104-62**	ATT ACT GCA AGT GCA AAT TTT TTT TTT TGA GGG GCA CCG TG
**core-91-18**	ATT ACT GCA AGT GCA AAT TTT TTT TTT AAG AAA TAT ACT TCA GGT
**core-82-31**	ATT ACT GCA AGT GCA AAT TTT TTT TTT ATA AAT CGA ATC GTA GAC TGG AAA AAC CTA A
**core-105-51**	CAG TAT CAG TAT CAG TAT TTT TTT TTT TAT GTA ATT TAT CAG ACG CTG TGT A
**core-96-27**	CAG TAT CAG TAT CAG TAT TTT TTT TTT AAA GCC TTC GTA ATT CA
**core-93-20**	CAG TAT CAG TAT CAG TAT TTT TTT TTT TTG TTT GAG AAG GAG GAA
**core-85-32**	CAG TAT CAG TAT CAG TAT TTT TTT TTT CAC CGC CTC CGA AAC GAC
**core-102-21**	CAG TAT CAG TAT CAG TAT TTT TTT TTT CAT TCA GCG ACA GTC TTA
**core-99-6**	CAG TAT CAG TAT CAG TAT TTT TTT TTT ATT TTC GCT CAA CAC AGA
**core-92-12**	CAG TAT CAG TAT CAG TAT TTT TTT TTT CCG TTC TGA GAA AGT GAC AAC AGG TG
**core-84-1**	CAG TAT CAG TAT CAG TAT TTT TTT TTT CGC GTT TAT TAT AGT AG
**core-122-77**	TGG ACT TAT ACT TGG ACT TAT TTT TTT TTT ACT ATC AAA AAT AGT GTT
**core-113-35**	TGG ACT TAT ACT TGG ACT TAT TTT TTT TTT AGT ACC GAT AAG TAT CAG AAG AAT GAC C
**core-109-65**	TGG ACT TAT ACT TGG ACT TAT TTT TTT TTT GAT ATT CTG AAC GGG GGC CTC GCC
**core-110-45**	TGG ACT TAT ACT TGG ACT TAT TTT TTT TTT TAG TAA ATT TCA ACA AAG
**core-108-61**	TGG ACT TAT ACT TGG ACT TAT TTT TTT TTT ACA CTA ACG GAG ATT AAA
**core-111-51**	TGG ACT TAT ACT TGG ACT TAT TTT TTT TTT GTC GCT GCC GAC AAG CCG GAG AAT GCC T
**core-52-107**	AAT GAC TTG AGA AAC CAT TTT TTT TTG TAC ACA TCA TTG TAA TC
**core-73-106**	CGA GAA ACG ACA GTT ACT TTT TTT TTG TAC ACA TCA TTG TAA TC

Table S2. Sequences of poly-thymine (pT) passivated strands in cylinders. Names denote both the function of the staple strand and its position according to the line numbers in Figure S5: [function- 5' position – 3' position].

Name	Poly-thymine passivated strands 5'→3'
**pT-21-22**	TTT TTT TTT TTT TTT CTG ATA AAT TGT TGA CCA ACT TTG TTT TTT TTT TTT TTT
**pT-37-40**	ACT TTC CAA CAT TTT GAT TAG CTC AAC ATG TTT TAA ATT TTT TTT TTT TTT T
**pT-45-8**	TTT TTT TTT TTT TTT GGC GCG AGC TGA AGT TTC AGC GGA TTT TTT TTT TTT TTT
**pT-27-28**	TTT TTT TTT TTT TTT TAA TCA TTG TGA AAC GAA CTA TTT TTT TTT TTT TTT
**pT-36-1**	TTT TTT TTT TTT TTT AGA ACG GGT ATT CGG AAC CTA TTA TTT TTT TTT TTT TTT
**pT-11-12**	TTT TTT TTT TTT TTT AAG GCT CCA AAA AAC AGC TTG ATA TTT TTT TTT TTT TTT
**pT-74-73**	TTT TTT TTT TTT TTT GTA AGA ATA CGT GCC CTA AAA CAT TTT TTT TTT TTT TTT
**pT-52-51**	TTT TTT TTT TTT TTT TTT TTT AAT CCT TAG AAT TTT TTT TTT TTT TTT

**pT-79-76**	TTT TTT TTT TTT TTT GTG TTG TTC CAG TTT GCC CGA GAG CAG GCG AAG C
**pT-38-37**	TTT GTC TTT CCT TAT CAT TCC ATT TTT TTT TTT TTT T
**pT-6-43**	TTT TTT TTT TTT TTT CGC CAG CAT CCA TAT TTA TTT TTT TTT TTT TTT
**pT-0-34**	TTT TTT TTT TTT TTT TTC TGA AAC ATG AAA GTA TAG AAC GCC CAA
**pT-37-36**	TTT TTT TTT TTT TTT TTA AGA GGA AGC CCG AGA TT
**pT-71-72**	TTT TTT TTT TTT TTT CAC AAT TCC ACA TGC GCT CAC TTT TTT TTT TTT TTT
**pT-34-33**	TTT TTT TTT TTT TTT TCA TTA CCG CGC GAG GCG TTT TAG TTT TTT TTT TTT TTT
**pT-56-16**	TTT TTT TTT TTT TTT ACA GTA CCT TTT ACA TAG ATG AAA ATC
**pT-18-59**	TTT TTT TTT TTT TTT TGT TAG CAA CCT ACC ATA TTT TTT TTT TTT TTT
**pT-4-5**	TCC ATG GAA AGC GCA GTC TTT TTT TTT TTT TTT T
**pT-61-20**	TTT TTT TTT TTT TTT TTT CAT CAA CAT TTG TAT CAT CGC TTT TTT TTT TTT TTT
**pT-7-7**	TTT TTT TTT TTT TTT CTT TCC AGA CGT
**pT-18-19**	AGA TCC TTA TTA CGC AGT ATT TTT TTT TTT TTT T
**pT-61-62**	TGT GTA ATG GGA TAG GTC ACG TTT TTT TTT TTT TTT T
**pT-41-4**	TTT TTT TTT TTT TTT TAT GCA ACT AAA TCG TCA CCA GTA TTT TTT TTT TTT TTT
**pT-3-38**	TTT TTT TTT TTT TTT AGA ACC GCC ATT AGA GAG TTT TTT TTT TTT TTT
**pT-17-59**	TTT TTT TTT TTT TTT TTG AGG ACT AAA GAC TTT TCA CCA ACT TTT
**pT-20-61**	TTT TTT TTT TTT TTT AGA AGG AAA TGA TTA TCA TTT TTT TTT TTT TTT
**pT-30-29**	TTT TTT TTT TTT TTT ATC CTG AAT CTT ACA GCC ATA TTA TTT TTT TTT TTT TTT
**pT-16-58**	ACG GAT AAT CCG TTA ATA TTT TGT TAA AAT TTT TTT TTT TTT TT
**pT-2-36**	TTT TTT TTT TTT TTT GGG GTC AGT GCC TTG AGT ACC TAT TTA AAC
**pT-51-52**	TTT TTT TTT TTT TTT TAT TTT AAA TGC ACA GTC AAA TCA TTT TTT TTT TTT TTT
**pT-43-6**	TTT TTT TTT TTT TTT TAG ATT TAG TTT AAG TTT TGT CGT TTT TTT TTT TTT TTT
**pT-58-18**	TTT TTT TTT TTT TTT TCA AAA TTA TTT GCA CGT TAG AAA CGT
**pT-39-3**	TTT TTT TTT TTT TTT TAC CTT TAA TTG CTC CGG TCA GGA CCC
**pT-35-34**	TTT TTT TTT TTT TTT CTT TAA ACA GTT CAG AGA A
**pT-76-75**	TTT TTT TTT TTT TTT AGA TTC ACC AGT TGA CCT GAA AGC TTT TTT TTT TTT TTT
**pT-13-14**	TTT TTT TTT TTT TTT CCG ATA GTT GCG AGG CTT GCA GGG TTT TTT TTT TTT TTT
**pT-49-50**	TTT TTT TTT TTT TTT CAT TAT GAC CCT AGA ACC CTC ATA TTT TTT TTT TTT TTT
**pT-13-15**	AAT ACG GAA AAT TGA GGG AGG GAA GGT TTT TTT TTT TTT TT
**pT-10-47**	TTT TTT TTT TTT TTT GCG TCA GAC ATA TAT TTT TTT TTT TTT TTT TTT
**pT-48-50**	TTT TTT TTT TTT TTT ACC TCC GGC TTA GGT TAG AGA CTC ATA
**pT-24-23**	TTT TTT TTT TTT TTT ATT GAG CGC AAG AAA CAA TTT TTT TTT TTT TTT
**pT-63-64**	TTT TTT TTT TTT TTT TGG TGT AGA TGG CAG CTT TCC GGC TTT TTT TTT TTT TTT
**pT-4-41**	TTT TTT TTT TTT TTT CTG AAT TTA CAG ACG ACG TTT TTT TTT TTT TTT
**pT-9-47**	TTT TTT TTT TTT TTT GTG AGA ATA GAA AGG AAC AGT TGA AAT TAG
**pT-36-0**	GCA TCG AGA GCT CAG TAC CAG GCG GAT TTT TTT TTT TTT TT

**pT-12-11**	TTT TTT TTT TTT TTT CAC CAT TAC TTG CCT TTA TTT TTT TTT TTT TTT
**pT-31-32**	TTT TTT TTT TTT TTT TTA CGA GGC AAA AGA AGT TTT TTT TTT TTT TTT
**pT-36-35**	CAA TTT ATT TTC ATC GTA GGA ATT TTT TTT TTT TTT T
**pT-62-63**	TTT TTT TTT TTT TTT GTT TGA GTA ACA TTA TGT TA
**pT-25-26**	TTT TTT TTT TTT TTT AAC GTA ACA AAG TAA TTT CAA CTT TTT TTT TTT TTT TTT
**pT-72-71**	TTT TTT TTT TTT TTT CGC CAT TAA AAA AAC ACC GCC TTT TTT TTT TTT TTT
**pT-22-21**	TTT TTT TTT TTT TTT TGA AAT AGC AAA GTT ACC TTT TTT TTT TTT TTT
**pT-3-2**	TCA CAG AAC CGC CAC CCT CTT TTT TTT TTT TTT T
**pT-68-67**	TTT TTT TTT TTT TTT ATC AAA CCC GGT TAT CTA TTT TTT TTT TTT TTT
**pT-6-7**	CAG GCC ACC ACC AGA GCC GCT TTT TTT TTT TTT TT
**pT-8-9**	CCT CAC CGG AAC CAG AGC CTT TTT TTT TTT TTT T
**pT-16-57**	TTT TTT TTT TTT TTT TTT TGT CAC TAT ACA GTA TTT TTT TTT TTT TTT
**pT-54-55**	TAC TCG CGC AGA GGC GAA TTT TTT TTT TTT TTT T
**pT-1-36**	TTT TTT TTT TTT TTT TAA GTG CCG TCA AAA AGA TTT TTT TTT TTT TTT
**pT-66-65**	TTT TTT TTT TTT TTT AAA TAT CTT GGA TTT AGA TTT TTT TTT TTT TTT
**pT-57-16**	TTT TTT TTT TTT TTT TGT ACC CCG GTT GCT ACA GAG GCT TTT TTT TTT TTT TTT
**pT-4-42**	GTT GTA CGG TTC CCA ATT CTG CGA ACG AGT TTT TTT TTT TTT TT
**pT-55-56**	TTT TTT TTT TTT TTA GGC TAT CAG GTA ACT AGC ATG TCA ATC ATA TTT TTT TTT TTT TT
**pT-59-18**	TTT TTT TTT TTT TTT TTC GCA TTA AAT CTA AAA CGA AAG TTT TTT TTT TTT TTT
**pT-64-63**	TTT TTT TTT TTT TTT AGT ATT AGA ATT TTA AAA TTT TTT TTT TTT TTT
**pT-33-34**	TTT TTT TTT TTT TTT TTT GCC AGA CCT CAA ATG TTT TTT TTT TTT TTT
**pT-40-4**	TTT TTT TTT TTT TTT ACA ATA AAC AAC ATG TTT CTG TCC CGT
**pT-15-16**	TTT TTT TTT TTT TTT AGT TAA AGG CCG AGC A
**pT-8-45**	TTT TTT TTT TTT TTT ACC ACC GGA CAT AAT TAC TTT TTT TTT TTT TTT
**pT-38-3**	TTT TTT TTT TTT TTT TAA TAT CCC ATC ATA AGT TTT AAC TTT TTT TTT TTT TTT
**pT-23-24**	TTT TTT TTT TTT TTT AAA GAG GAC AGA ATT ACC CAA ATC TTT TTT TTT TTT TTT
**pT-47-10**	TTT TTT TTT TTT TTT CAA GGC AAA GAA ATC TCC AAA AAA TTT TTT TTT TTT TTT
**pT-73-74**	TTT TTT TTT TTT TTT TGC CCG CTT GGG CGC CAG TTT TTT TTT TTT TTT
**pT-47-48**	CAA AAA TCG GTT GTA CCA AAA ATT TTT TTT TTT TTT T
**pT-65-66**	TTT TTT TTT TTT TTT ACC GCT TCT GGT TTC GCT ATT ACG TTT TTT TTT TTT TTT
**pT-50-49**	TTT TTT TTT TTT TTT CCT TGA AAA ACC TTT TTA TTT TTT TTT TTT TTT
**pT-16-17**	AAT CCA CGG AAT AAG TTT ATT TTT TTT TTT TTT T
**pT-29-30**	TTT TTT TTT TTT TTT ACG GAA CAA CAA AAG GAA TTT TTT TTT TTT TTT
**pT-42-6**	TTT TTT TTT TTT TTT ACA ACG CCA ACA TGT AAG AAT CGT GA
**pT-32-31**	TTT TTT TTT TTT TTT CGA ACC TCC CGA AGC TAC AAT TTT TTT TTT TTT TTT TTT
**pT-43-44**	ATT TAG CTA TAT TTT CAT TTG GTT TTT TTT TTT TTT T
**pT-19-19**	TTT TTT TTT TTT TTT AGG CAA AAG AAT

**pT-5-43**	TTT TTT TTT TTT TTT CAA ACT ACA ACG CCT GTA GCG ATC TAG ACC
**pT-77-78**	TTT TTT TTT TTT TTT TTT GCC CCA TAG GGT TGA TTT TTT TTT TTT TTT
**pT-44-8**	TTT TTT TTT TTT TTT TAG AAA AAG CCT GTT TCC GGA ATA CCG
**pT-34-79**	TAG AAA ACC GTC TAT CAA GCC ATT TTT TTT TTT TTT T
**pT-46-10**	TTT TTT TTT TTT TTT AGT TAA TTT CAT CTT CTT TTC AAT GT
**pT-70-69**	TTT TTT TTT TTT TTT TGC AAC AGT ACC TCA AAT TTT TTT TTT TTT TTT
**pT-40-39**	CTA AAG TCC TGA ACA AGA AAA ATT TTT TTT TTT TTT T
**pT-54-53**	TTT TTT TTT TTT TTT TAT TCA TTT TGA ATT ACC TTT TTT TTT TTT TTT
**pT-60-61**	TTT TTT TTT TTT TTT GAT GAT GGC AAT TCA TAT A
**pT-75-76**	TTT TTT TTT TTT TTT GGT GGT TTT CCA CGC TGG TTT TTT TTT TTT TTT
**pT-78-77**	TTT TTT TTT TTT TTT TTG CAA CAG GAA ATT TAC ATT GGC TTT TTT TTT TTT TTT
**pT-53-54**	TTT TTT TTT TTT TTT CCA TCA ATA TGA AGA GAT CTA CAA TTT TTT TTT TTT TTT
**pT-26-25**	TTT TTT TTT TTT TTT ATA ACA TAA CAG AGG GTA TTT TTT TTT TTT TTT
**pT-45-46**	GTG ATC CAA TAA ATC ATA CAG GTT TTT TTT TTT TTT T
**pT-28-27**	TTT TTT TTT TTT TTT TTT ATC CCA ATC ACA GAG AGA TTT TTT TTT TTT TTT
**pT-59-60**	GTT TGG CCT TCC TGT AGC CAG CTT TTT TTT TTT TTT T
**pT-69-70**	TTT TTT TTT TTT TTT TTG CAT GCC TGC TTG TTA TCC GCT TTT TTT TTT TTT TTT
**pT-67-68**	TTT TTT TTT TTT TTT CCA GCT GGC GAA CCA GTG CCA AGC TTT TTT TTT TTT TTT
**pT-14-13**	TTT TTT TTT TTT TTT TAA ATA TTG CAC CAG TAG TTT TTT TTT TTT TTT

Table S3. Sequences of connector overhangs c1. Names denote the position of the connector in the folding map in Figure S5, with the line number being where the overhang protrudes from the origami (here: 5’).

Connector overhangs c1	
Name	**Sequence 5’→3’**
**c1-72**	1 2 3 4 5<br>GTAGTACTA GATTA GTGTAG TTAGTTAG GTGTAATA TTT TTT CGCCATTAAAAAAACACCGCC TTT TTT
**c1-68**	GTAGTACTA GATTA GTGTAG TTAGTTAG GTGTAATA TTT TTT ATCAAACCCGGTTATCTA TTT TTT
**c1-64**	GTAGTACTA GATTA GTGTAG TTAGTTAG GTGTAATA TTT TTTAGTATTAGAATTTTAAAA TTT TTT
**c1-40**	GTAGTACTA GATTA GTGTAG TTAGTTAG GTGTAATA TTT TTT ACAATAAACAACATGTTTCTGTCCCGT
**c1-44**	GTAGTACTA GATTA GTGTAG TTAGTTAG GTGTAATA TTT TTT TAGAAAAAGCCTGTTTCCGGAATACCG
**c1-48**	GTAGTACTA GATTA GTGTAG TTAGTTAG GTGTAATA TTT TTT ACCTCCGGCTTAGGTTAGAGACTCATA
**c1-0**	GTAGTACTA GATTA GTGTAG TTAGTTAG GTGTAATA TTT TTT TTCTGAAACATGAAAGTATAGAACGCCCAA

**c1-14**	GTAGTACTA GATTA GTGTAG TTAGTTAG GTGTAATA TTT TTT TAAATATTGCACCAGTAG TTT TTT
**c1-4**	GTAGTACTA GATTA GTGTAG TTAGTTAG GTGTAATA TTT TTT CTGAATTTACAGACGACG TTT TTT
**c1-30**	GTAGTACTA GATTA GTGTAG TTAGTTAG GTGTAATA TTT TTT ATCCTGAATCTTACAGCCATATTA TTT TTT
**c1-22**	GTAGTACTA GATTA GTGTAG TTAGTTAG GTGTAATA TTTTTTTGAAATAGCAAAGTTACC TTT TTT
**c1-18**	GTAGTACTA GATTA GTGTAG TTAGTTAG GTGTAATA TTTTTTTGTTAGCAACCTACCATA TTT TTT
**Cy5-c1-78**	Cy5/T GTAGTACTA GATTA GTGTAG TTAGTTAG GTGTAATA TTT TTT TTG CAA CAG GAA ATT TAC ATT GGC TTT TTT
**Cy5-c1-26**	Cy5/T GTAGTACTA GATTA GTGTAG TTAGTTAG GTGTAATA TTT TTT ATA ACA TAA CAG AGG GTA TTT TTT
**Cy5-c1-56**	Cy5/T GTAGTACTA GATTA GTGTAG TTAGTTAG GTGTAATA TTT TTT ACA GTA CCT TTT ACA TAG ATG AAA ATC
**Cy5-c1-4**	Cy5/T GTAGTACTA GATTA GTGTAG TTAGTTAG GTGTAATA TTT TTT CTG AAT TTA CAG ACG ACG TTT TTT

Table S4. Sequences of connector overhangs c1*. Names denote the position of the connector in the folding map in Figure S5, with the line number being where the overhang protrudes from the origami (here: 3').

Connector overhangs c1*	
Name	**Sequence 5'→3'**
**c1*-72**	9<br>TTT TTT CAC AAT TCC ACA TGC GCT CAC TTT TTT TAG CTA C GTATCTTCCAT
**c1*-68**	TTT TTT CCA GCT GGC GAA CCA GTG CCA AGC TTT TTT TAG CTA C GTATCTTCCAT
**c1*-64**	TTT TTT TGG TGT AGA TGG CAG CTT TCC GGC TTT TTT TAG CTA C GTATCTTCCAT
**c1*-40**	ACT TTC CAA CAT TTT GAT TAG CTC AAC ATG TTT TAA A TT TTT TAG CTA C GTATCTTCCAT
**c1*-44**	ATT TAG CTA TAT TTT CAT TTG G TTT TTT TAG CTA C GTATCTTCCAT
**c1*-48**	CAA AAA TCG GTT GTA CCA AAA A TTT TTT TAG CTA C GTATCTTCCAT
**c1*-56**	TTT TTT AGG CTA TCA GGT AAC TAG CAT GTA ATC ATA TTT TTT TAG CTA C GTATCTTCCAT
**c1*-78**	TTT TTT TTT GCC CCA TAG GGT TGA TTT TTT TAG CTA C GTATCTTCCAT
**c1*-0**	GCATCGAGAGCTCAGTACCAGGCGGATTTTTTTAGCTA C GTATCTTCCAT
**c1*-14**	TTTTTTTTTCCGATAGTTGCGAGGCTTGCAGGGTTTTTTAGCTA C GTATCTTCCAT
**c1*-8**	TTTTTTTTGGCGCGAGCTGAAGTTTCAGCGGATTTTTTAGCTAC C GTATCTTCCAT
**c1*-4**	TTTTTTTTTATGCAACTAAATCGTCACCAGTATTTTTTAGCTAC C GTATCTTCCAT T
**c1*-30**	TTTTTTTTACGGAACAACAAAAGGAATTTTTTAGCTA C GTATCTTCCAT

**c1*-26**	TTTTTTTTTAACGTAACAAAGTAATTTCAACTTTTTTTTTAGCTACGTATCTTCCAT
**c1*-22**	TTTTTTTTTCTGATAAATTGTTGACCAACTTTGTTTTTTTAGCTACGTATCTTCCAT
**c1*-18**	TTTTTTTTTTTCGCATTAAATCTAAAACGAAAGTTTTTTTAGCTACGTATCTTCCAT

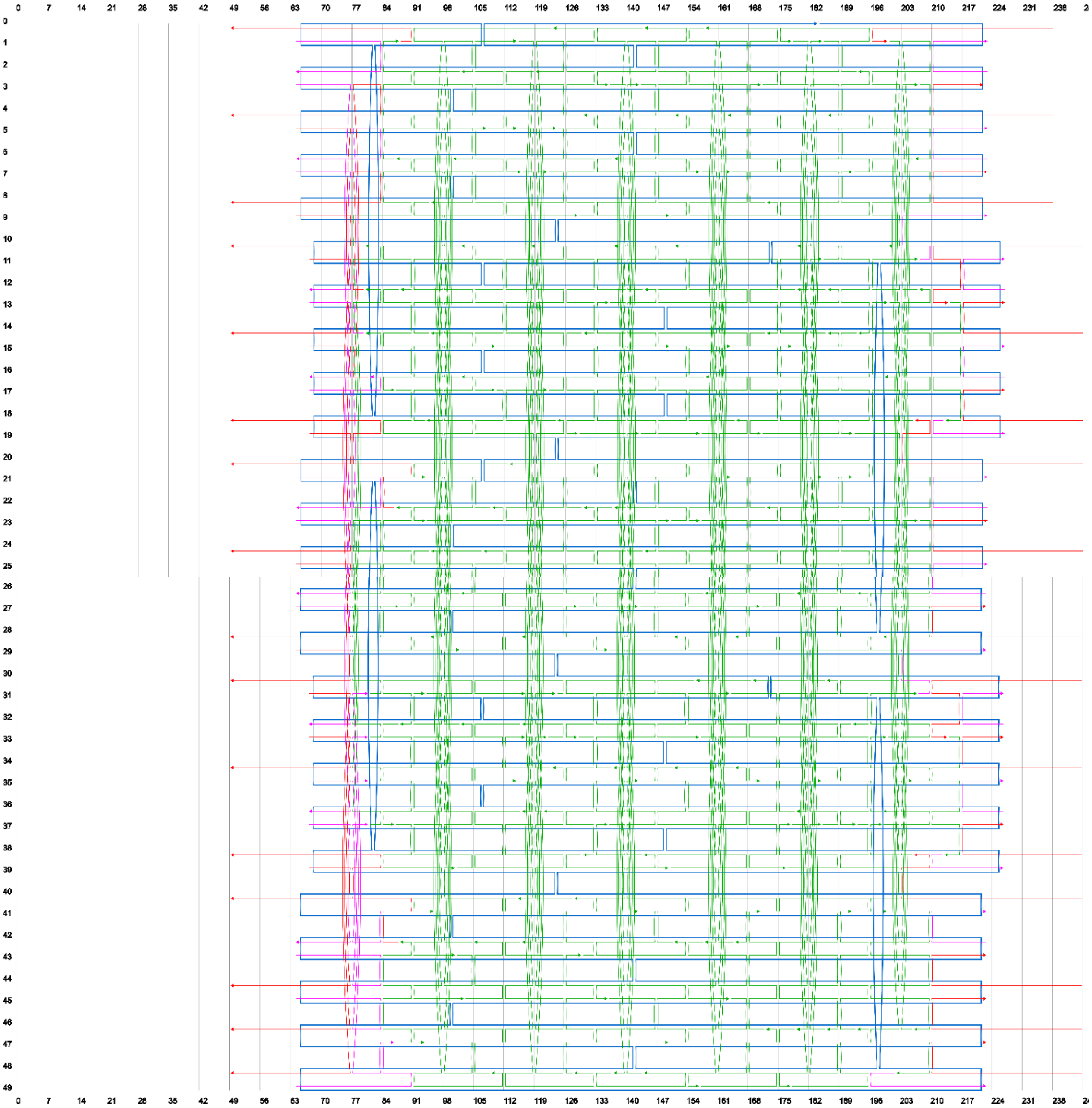

Figure S7. 3D DNA origami nanocuboid folding map. Scaffold is depicted in blue, core staple strands in green, pT passivated strands in pink and connector overhangs in red (16 connectors per side).

Table S5. Sequences of core staples in cuboids. Names denote both the function of the staple strand and its position according to the line numbers in Figure S7: [function- 5' position].

Name	Core staples 5'→3'
**Core_0**	TTG CTT TGA CGA GCA CGC ACG TAA AAC AGA AAC ATA TCA GAA T
**Core_0**	GCT ACA GGG CGC AGA GCG GCA ATT CTC ATA TTG AT
**Core_0**	GCG CCA AAA GAG TTG GTG CAT CGT AGA GAG ATG CA
**Core_1**	TGA GGA CCG TAA GTT TGA CCG GAG AGG TAA AGT AT
**Core_1**	TGT TCC AGC AGG CGA AAA CAG TGA GCA TTC GCA AC
**Core_1**	CAA CGT GGG ATA TCG GCG TTC TAG GAA AGG CTC AT
**Core_1**	TGC TGA TTG TTC CAG AAG ACA AAA TAC GCA AGA TC
**Core_2**	GGG ATT TAT CGG CCC GCC AGC TGG CGC AGA AAC
**Core_2**	CGG TAC GCC AGT CCA TCA TTA AAT CTT TAC AAT TC
**Core_2**	TGC CCC AGT TTA ACC GTC TAT CAC GCT GCG CGG GT
**Core_3**	GGT TGT GCG GGA AGT TGG ACC AAA AAA AAT CAT CA
**Core_3**	TCA CTC CTG TTG AGA TAG TAA CCA CCA CAC CCG CC
**Core_3**	CCG GAG CCG GAA GCA TAG TGA AAT AAA CGT AGG AT
**Core_3**	GTA TCG TTG CGC TCA CTG TTA CCG CGA GGA AGG GC
**Core_4**	GCT TGG GGA GAG GAG GCC TTA GAA TCG TAC TAT GG
**Core_4**	CCT GGG GTG CCA TGG TCA CCA GTC CAA AAA GAT AG
**Core_4**	ATT GTG GGC GCA AAT CGG TAT AAA TTT AAT GCG CC
**Core_4**	TAA CTC ACA ACC GAG CGA TAG ACT TTC TAA CAG
**Core_5**	GTA ACT TTA GGT CAA TCA CCT CCG GCC TTT TGT AG
**Core_5**	CTT GCT GTC GTT TAA TGA TAG ACA GTT GTA TAA CG
**Core_5**	AGA AAC GCT CAT GGA AGA TG
**Core_6**	TGA CGC TCA TAA TAA ACA GTG CCC GGT CAG TAT
**Core_6**	TCT AGT AGA ACG TTG TAG AGT CTG AAT CCT GAA CC
**Core_6**	TGC AAG CGG TGG TTA AAC GCA GCC TAG ACC GGA TA
**Core_6**	ATA CCG GGG GTG TGG TGC ATC GAC TTC AAA TGC TT
**Core_7**	CCA GGC TGG TAA TGG GTA GCC ATC CAC GTC AGC GT
**Core_7**	TAC CCT TAC ACT GGT GTG ATT AGT CTT TAA AAG AA
**Core_7**	CCG TTC CGG CCG TTT TTA GG
**Core_7**	ACT CTT TCT GCT TCC TGT AAG TGT AAC AGC TGG TT
**Core_7**	AGA CCC TTT AGT CCT CAT ATT CGA GAG AAG TTC AAC A

**Core_8**	TTC TAG ACA ATC GCC ATT AAA AAT AGG TGA GGA CG
**Core_8**	TGC GCG CAG TGG TTC TTC CGT AAT CTA ATG AGT TT
**Core_9**	TGC CAA CAT CCT GCA GCC ACA GGA AAA CAA TAC CC
**Core_9**	TGG TAA GGT TTC CCC TGC CCT CAC ACC CGG GTT TA
**Core_10**	CAG AAG CGG ACT TGT AGA CAC GCA ACC AGC TTC CC
**Core_10**	CAG CAG CAA CCG TCG CTG GAT GCT GGT CAG TTG TT
**Core_10**	ACG AGA ATA CGT GGC ACG GCC AAC CGA CCA GAT CG
**Core_10**	AAC TGA TAG CCC TAC CGG TCA AAC GCG CAA ACC CAG CAC TAG AAA A
**Core_10**	GGT GGA GCA CAT GAT GAA TGC CGT GGT GAA GGA TA
**Core_10**	CTT TAC CCT GAA AAA AGA ATA AAA AAA ACA GCC AG
**Core_10**	ACA TAT TTT TGA ATG GCT TTC AGC AGG CAT CAA TAC CTA GGC
**Core_11**	CAT CCT ATT ATG AGG TGT CCA GCA TGT ATG AGT GC
**Core_12**	CTG AAC CTT GCA GAG CCG ATT AGA CCT TTG CCA TT
**Core_12**	AAA AAA GCG GAG ACT TCA AAT AGT AAA ATC ACC GGC AT
**Core_12**	TAA ACC GGT GCC TTT GCT CGT CAT AAA CGG CAG GT
**Core_12**	CCC GGC GCC TGC CGG GTC ACT GTT GAC GGC TGA GT
**Core_12**	TGT ACT GCG GCC GGG CGC GGT TGC GCA GCG GGG GT
**Core_13**	TGC GGA AAC ACG CCA CGG CAC CGC AGA TCG CCC CG
**Core_13**	CAA ATT CCC ACA ACT AAG AAC TGG GCG ACT GTA GC
**Core_13**	AAA TTC TGA GAT TGC GGA ACG GAA CTC AAC TAA AA
**Core_14**	CGC CCC AAG CTA CCA GGC ACG ACA GAC AAA CGT TC
**Core_14**	TTT ATA GCT GTC AGC ACG CGT GCC TTC ACT GCT AA
**Core_14**	AAT TGT TGT AAC ATT CAG ATC TGC CAT GGG ATA AA
**Core_14**	GAT TTG AAC CTT ATA ACT TTT TTC ATA TTT AAA GT
**Core_14**	ATC TGG CGA TTC CTC TTC ATT ATC AAT CAA TAT TT
**Core_15**	GGT TGG GAA GGA TGG GCG TAG ATC ATT AAC CAA TA
**Core_15**	GGG GGA TGT TAA TAC TAG AGT CAT GTT TAT AAA AG
**Core_15**	GAC GAT TAA GCG CGA GCT ATC AAC GAA CGA GTC AG
**Core_15**	CGA CTG TGA GAT CGA ATT GCG TCC GTG AGC CTA TC
**Core_16**	TGT TTT CCC AGG CAT AAA AGC TAT AAT TCA GTG AA
**Core_16**	CGT ACG CTG CAG CCA GCA AAT TAA CGA ACT CAA TA

**Core_16**	GAA TTC AGA GGC AAG GCA ATT CTA AAC CGG AGA GA
**Core_17**	CGG AGC TAT TAA ACG CGC TCC AGT CGG GAA ACC TG
**Core_17**	CAT GGA TTA TTG CTT TGA ATA CCA TCG CG
**Core_17**	ATC AGG AAG GGA ACA ATA ACG GAT TGC ACC CAT TA
**Core_18**	AAT TTT TGC GAA TTT CAA AT
**Core_18**	CTC GAG TCC AAG CGG TCC ACG CTG ATT GCC CGC CG
**Core_18**	ATC CTT TCC TCG GAT TAA A
**Core_18**	TCA CAT AGC CCT GAT GGT GGT TCC GCA GGG TGA AC
**Core_19**	CTG TAG CCA GCA AAG CCC GTA AAA CAG ACA GCA CT
**Core_19**	GTT AAA TCA GCT AAA ATT CAA AAG ATT ATC CGA GC
**Core_19**	TTT TGA TGA TGG GTC CCT CAA AAA GCT AAA CAG GC
**Core_19**	GGA ATT GTA AAC TGA GAG GAG GAC TAA AGA ATA AT
**Core_19**	AAT AAT TCG CGT TGT ATA GAG AAT CAG CAA CGA CG
**Core_20**	CAG AGG TGC TAT TTT CGC CTG ATA CTT CTA AAT TAT GAA
**Core_21**	GAA CAT TAA TGG GGG ACG AAA GCG CAC GGG CAA AG
**Core_21**	AAT CCC GAC TTG CCG TTT CCT GAA CGA GTT AAG CC
**Core_21**	AAT CCA AAA ACA GGA AGA TCT GGC CGC GGA TTT GT
**Core_21**	GGA GTA TTT TTA CCG TGC GCT GCG CGT TTT TCT GAG C
**Core_22**	TTA CGT CGA ATT ATT CAT CGA GGC GGG AAT CAG TT
**Core_22**	GAA AGA ACC CTC GCC TGA CGC AGA CAC CGA AGC AG
**Core_22**	ACA TAC CTG AGC GCA TTA AAA CAA AAA GTT TTT A
**Core_22**	TAG CCA AAC AAA GCA AAT ATT TAA ACG CCA TCA GG
**Core_23**	ATG CTA CAA CGG AAC CGA AGC CCG GTT CGG AAC CT
**Core_23**	AAA TCT ACA AAT CAT TGC CGT TAA TAT TTT GTT CA
**Core_23**	AAC CTC ATT CCA TCA ATA AGT TAC CTG ATT AAG AGG T
**Core_23**	GAC CAT ACA GGT GGA GCA TCG GCG TGT TAT CGG TC
**Core_24**	TTG TGT AAC GCA GGA ATT CAG GAT CGT TGA GCC AT
**Core_24**	GGG AGA AGC ACA ATT CAC AAC TAA ACT ATC CAT TT
**Core_24**	TGT AGC TTA GAT CCT GAA AAG AGA ACC TTA TTA AA
**Core_26**	CAT TAT TGC TGC TAC GTT CGC CAA AAG AGC CAT AC
**Core_26**	GTT ACA AAT ATC CTG CAA AGT TAA CAA TCG GGA CA

**Core_26**	ATT TGC TCA ACC AGG ACG ACC AGA GAC CCT CAC GT
**Core_26**	CTG CTG GCA TCA AAG AAT AGG GTG ACT GAT AAG GT
**Core_27**	CTT AGC GAA CCC CGG CCA CTG GTC TGC ACC GTC GG
**Core_27**	AAC ATA TGT AAG CGA TAA ATC GTC GTG AGT GTT TG
**Core_27**	ATT TGA CTA CCA TTT ATC ACA TTA TAT TTT TAC AA
**Core_27**	TGT AAG TTT GAT TGG GGC AAT AAA GAT GTG TAG GG
**Core_28**	TAA GAT AGC GTT TGC CAT CTT TTC CGG AAC CCT CA
**Core_28**	TTT AAA CGG AAC GTG CCA AGC CGC ACT CAC GGC GG
**Core_28**	TCA GAA TAA ACT AAT TAC TAG AAA AGA TCA GTT GAG A
**Core_28**	TTT AAT AAA TAT TAG TAT CAT ATG CGC TCA ACC AA
**Core_28**	CAA AGA GCT TAT CGC AAA CCT GTT TGC TAA ATT TT
**Core_29**	GTT AAA TAA GGA TTA GAG AGT ATA TAT TAA CAA A
**Core_29**	GCG ATT ACC AGA CGA CGA ACC ACA TAA CAT TAT TC
**Core_30**	CTA TCA TAA TAC ATA AAA TAA AAA AAG GCT TGC
**Core_30**	AGT AAG AGT TGC CAG AGG GGG TAA TAT CGT GC
**Core_30**	ATC AAA ATG TTT AGA CTG GAG GAA GAA ATA TGA TT
**Core_31**	ATT AAA TCA ACC GGA AAA CCA AAA AGC AAA GTC
**Core_31**	AAT TAC GAG GAT TGG GAA ACA CCA GTA ACA AAA AC
**Core_31**	TTG CCA AAG CCA TTT GGG AAT TAG AGC CAG TAT GG
**Core_31**	AAC GTT ATA CAT ACC GAC CGT GTG GTT AAT TAG AA
**Core_31**	AGG GTT CAA CCT ATC ACC GTC ACC GAC TTG AGA CA
**Core_32**	CGC CCA GTA ATC AAG AAA GAA ACC AAA GAA CGC AA
**Core_32**	GCC AAA CCT TTA ATT GCT CTT AGG TGC TGA GAT TTG C
**Core_32**	AAA GAT TCA GAT ACC GAC CAA CAA TGT CTC CAT GT
**Core_32**	TTA TGG GCT TTA TTC ATA ACG GTG TGA CCC CTA AT
**Core_33**	CCT GCA GTA AGC CAC CAC CAG AGC CCA CCC TCA GG
**Core_33**	AGC AAC ATG TGA ACG CGT CAT CTT AAA CAG ACC GA
**Core_33**	GTA ATT ACA GGA TAA GAG CTC CAA CTC GTC TCG CTG CGC G
**Core_33**	GTT GGT AAT CGG AAT AAG TTT ATT ACC AGC TAG GA
**Core_33**	TGT CAT GCA GAA ACG AGG TAA ATT GGC CCA ATG TA
**Core_33**	CTT GTA ATC TAT AGG CTA TAC ACT AAC CTA ACC CT

**Core_33**	TGG TTA AAG CAG GTC AGA CGA TTG CCT TTA GC
**Core_34**	GCT ACA GAC GAT TTG ATG CAA TCA ATA CCG CCA CC
**Core_34**	TAA CCA TAA GGG AGA TTT ATC TTT TTC ATG AGA CA
**Core_34**	GCT CTT TTC ATC CAT TAG GAT AGC TCA GGC GGG AT
**Core_34**	CGC CAT AGT GAT TTT TAA ATA TCT GAT TGC CGG GT
**Core_35**	GAT GTA CCC AAG AAA AGG GAA CGA GGA CGC AGA AT
**Core_35**	GCG CTG ACA AGC TAA TAG ACA GTT GAC TTA AAT TG
**Core_35**	CAA TAA CAT GTT AAA TAA TGG TCG GCA ACA GTG GG
**Core_35**	TAC TAG TAA GCG AGT AAC AGT GCC CAG TGT ACA AA
**Core_35**	TTT GAG CGC GAC CAT TAA GCT ACA GCG CAT AAC C
**Core_35**	GTA AAT AAT AAA AAC ATA ATG CTG AAG CAT CGA GC
**Core_36**	AAG ATA AAA GAC CCT GGC TGC AAA AAA TAT TAT CC
**Core_36**	CGC GAC CTG CTC ATC GTA TTT TAG CCA ATC CAA ATA AGA TGT T
**Core_36**	CTT TAC AGA CTA CCA GGG AGA CTC CTC AAG AAT CC
**Core_36**	ACC AAA AGA GGC GAG AGG ACA TGA AAG TAT TAG AA
**Core_37**	ACG AAA TAG GAG CCA CCC TCA GAG CTG CTC AGC AG
**Core_37**	GCA AGC GGG AGA AAC AGC CAT ATT AAT AGC AGT AAC T
**Core_37**	TAC GCA GCG ATA TTC AAA TAG CAA AGC CAG TGA TG
**Core_37**	GCC ATC GGA ACG CTG AGG CTT GCA TCC AAA AAC AA
**Core_37**	GAA GGT ATT AAA TAT ATG CTA TTA TAG AAG TTC AA
**Core_37**	AAG CAT TCC ACA TAG CTA TCA CCG TAC TCA GGA CA
**Core_37**	GGT AAC ACT GAA GAA CCG CCA CCC TGT GCC GTA CA
**Core_38**	GAA TAG CAT GTT CAA CCC TCA GGA TTC TGG TTT CA
**Core_38**	AGG CAG TCA GGG GCT AAT CCT GAT TGC GAT CGT GC
**Core_38**	TTC TGA TAC CGA TAG TTG GAA TTT CTT TTC TGT AT
**Core_39**	GAC AAC AAC CAT TAT CAG AAA CAA CTT TCA ACC AC
**Core_39**	GAT ATG GAG CCT GCG GAG TGA GAA TAT ACC GTA AAA
**Core_39**	ATC CGA ACC TCA AGA TTA ATT TAA CGA ACA AAA CT
**Core_40**	CAG CTT TTT AAC GAT TAA GAA CGT TCA ATT CAA GAA AGA G
**Core_40**	TAA TTC CAG ACG TTA GTC CTC ATA TTA CCG CTG TCG
**Core_41**	GGG ACT GTA GCA AAT CAG GTA TCA TCA TAT ATC GG

**Core_41**	TTC ATT AAT TGT ATC GGT TCG CCC AAG GCT TTT CT
**Core_41**	AGG AAA AAG GCT CCA AAA ATT CGG TCG AGG GTG AT
**Core_41**	GAA CGT TTT GTC GTC TCG TCA AAA ATG AAA TTT
**Core_41**	CTA AAG GAA TCA AGC CCA GGC ACC AAA ACA CCG GA
**Core_42**	CAG TGA AGT TTA ACA AAG CTG AGT ACC TCA GAT CA
**Core_42**	CTA AAA CTT ATT TTT TCC TTA TTG CCT TTA TTT CAT AA
**Core_42**	AGC GGC AAG AAA CAA TGA TAA GAA ATA GCC GGA CG
**Core_42**	ACG CTT TTG CTC TTG CTT TCG AGG TCG CCG ACA GA
**Core_42**	AAC TAG GTT TAG TAC CGC TAA GTA TAC TGA CCG CT
**Core_43**	GCA AGA CAG CAA ATG AAT TAA A
**Core_43**	CAA TCG AAC AAA TCG GCT AGA TAA GTT AAG ACT GG
**Core_45**	TTA ATG CCC CCC ATG GCT CGA CAA TCG AAC TAT GG
**Core_45**	CAG TGC CTT AGA TAG CAA TAA GAT AAC GAT
**Core_45**	ATT AAC CGT TCA CGA GAA GAA AAA TAA TAT AAC GT
**Core_46**	AAT GAG AGG CTC GGA TAA CAG AAC CAC CCA TGG AA
**Core_46**	TCA TTT AAT TAT TTT AAA GTA CGG TTG AAT CCC CC
**Core_46**	TCT CGC ATT GAC AGG AGG AGC CGC CCC ACC ACA TA
**Core_46**	AGC GAG TAA ATT ACC AGT ATG TTT TCC CGA AAT TG
**Core_46**	ATT TTT CTG AAG TTG ATA CAC CCT CGT TTC GTA GT
**Core_47**	AGA ACG TCA TAT GCC TAT AAT AGG TGT ATC TTG GT
**Core_47**	ATA TAC GCA GTA CTG GCA AGA AGG AAA GAA TTA AA
**Core_47**	AGA AAC GCA AAG GCG ACA CTT AAT TAG CCT GTA GGC
**Core_48**	AAA GGA CAC CAA AGT TTT AAC GGC TTA TAA AGA TC
**Core_48**	AGG AAT GCA GAC CCT CGT GAG GCT TTT GCA AAC TT
**Core_48**	TTT TTG TCA ATA CAG GGT ATA AAC CCT TTT AAT A
**Core_48**	TTC ACA AAA TCA CCA GTA GCA CCA TCC ACC CTC AG
**Core_49**	CAC CAA TGA AAC CAT CGA CCC TCA GTT GAG GCC AG
**Core_49**	CAT TAG CAA GGC CGG AAA GAA CCG CGC CGC CAT GA

Table S6. Sequences of pT passivated strands in cuboids. Names denote both the function of the staple strand and its position according to the line numbers in Figure S7: [position of protruding end_function_line number].

Name	pT passivated strands 5'→3'
**Left_pT_0**	TAC TAA AGA AAT TGC GTA GAT TTT CAG GTT TTT TTT TT
**Left_pT_2**	TTT TTT TTT TTA ACG TCA GAT GAA TAA GAG AAG TGT TTT TAT AAT CAT TTT TTT TT
**Left_pT_4**	ACG TAG TAA AAG CAA TAC TTC TTT GAT TAG TTT TTT TTT
**Left_pT_6**	TAG TTG CCT GGA AAT GGA TTA TTT ACA TTT TTT TTT TT
**Left_pT_8**	CAG AGT CAC AAG AGA TAG AAC CCT TCT GAT TTT TTT TT
**Left_pT_10**	TTT TTT TTT CCT GAA AGC GTA AAC CAC CAG TTT TTT TTT
**Left_pT_12**	TTT TTT TTT GGC AGA TTC ACC CAG CAA ATG TTT TTT TTT
**Left_pT_14**	TTT TTT TTT TAA TAA CAT CAC ATA ATA CAT TTT TTT TTT
**Left_pT_16**	TTT TTT TTT AAC GCT AAC GAG CGT CTT TTA CCT TTG AGT AAC CGA
**Left_pT_16**	TTT TTT TTT GTG AGG CCA CCG TAT TAA TTT TTT TTT TTT
**Left_pT_18**	AAT TGA AGC CGC TAC AAT TTT ATC CGG GAG ATT TAC AGT AAC AGT ACT TTT TTT TT
**Left_pT_20**	TTT TTT TTT CTT TTA CAT CTG AAT CTT ACC TTT TTT TTT
**Left_pT_22**	TTT TTT TTT TAA AAG TTT TTT AAT GGA AAC TTT TTT TTT
**Left_pT_24**	TTT TTT TTT TTG AGG ATT ATT AAT TTT CCC TTT TTT TTT
**Left_pT_26**	TTT TTT TTT AAA AAT CTA ATG CAA ATC CAA TTT TTT TTT
**Left_pT_28**	TTT TTT TTT CAG AAG ATA CTG ACC TAA ATT TTT TTT TTT
**Left_pT_30**	TTT TTT TTT TAA TGG TTT GAA AAA TTC TTA TTT TTT TTT
**Left_pT_31**	TTT TTT TTT ATA CAT ACA TAA AGG TGG CGG AGG GAA GCC
**Left_pT_32**	TTT TTT TTT TCG CAA GAC AAA AAT TTA GGC TTT TTT TTT
**Left_pT_33**	TTT TTT TTT TAA CGG AAT ACC CAA AAG AAT GTT AGT TCG
**Left_pT_34**	TTT TTT TTT TTA GAA TCC TTG TCC CAT CCT TTT TTT TTT
**Left_pT_35**	TTT TTT TTT TAT CAG AGA GAT AAC CCA CAA CCG AGG CAT
**Left_pT_36**	TTT TTT TTT AGT ACA TAA ATC AAC CAA GTA TTT TTT TTT
**Left_pT_37**	TTT TTT TTT CAG GGA AGC GCA TTA GAC GGA GGG TAT CGA
**Left_pT_38**	GTC AGG AGA ATC CTT TAC AGA GAG AAC AAA ATG TTT CCA GAG CCT AAT TTT TTT TTT
**Left_pT_40**	TTT TTT TTT TTG CCA GTT ATA ACA TAA AAA TTT TTT TTT
**Left_pT_42**	TTT TTT TTT CCG CAC TCA ATT GAG CGC TAA TTT TTT TTT

**Left_pT_44**	TTT TTT TTT AAT TTA CGA GAA ACG CAA TAA TTT TTT TTT
**Left_pT_46**	TTT TTT TTT AGA GGC ATT CAA ACG TAG AAA TTT TTT TTT
**Left_pT_47**	TTT TTT TTT ACG GAA ATT ATT CAT TAA AGG TGA ATG ATT GAG AAC
**Left_pT_48**	TTT TTT TTT CCA GTA TAA AGG TAA ATA TTG TTT TTT TTT
**Right_pT_0**	TTT TTT TTT GTG GAC TCC AAC GTC AAA GGG CGA AAG GAA
**Right_pT_2**	TTT TTT TTT AGA GTT GCA GCA CTA TTA AAG AAC TTT TTT TTT
**Right_pT_4**	TTT TTT TTT CAC ACA ACA TAC CCT GGC CCT GAG TTT TTT TTT
**Right_pT_6**	TTT TTT TTT GGC CGT TTT CAC CGC TCA CAA TTC TTT TTT TTT
**Right_pT_8**	TTT TTT TTT CAC TCA ATC CGC CAG AAT GCG GCG TTT TTT TTT
**Right_pT_10**	TTT TTT TTT GAA TGA CCA TAA ATC AAC AGT TCT GGG CGG TTT C
**Right_pT_11**	TAA AAA ATC AGT CAT TGC AGG CGC TTT CGT TTT TTT TT
**Right_pT_12**	TTT TTT TTT GCC AGC AGT AGA AAA CGA TTT TTT TTT
**Right_pT_14**	TTT TTT TTT AAC CTC ACC TCA TTT GCC TTT TTT TTT
**Right_pT_16**	TTT TTT TTT AGC TTT CCG GGA ACG GAT TTT TTT TTT
**Right_pT_18**	TTT TTT TTT GAG TAA CAA ACT CCA GCC TTT TTT TTT
**Right_pT_18**	TCG AAC ATT AAA TGT GAG CTT TTT TTT T
**Right_pT_20**	TTT TTT TTT ACC CCG GTT GAT AAT CAG ATT TCA TCG ATT
**Right_pT_22**	TTT TTT TTT ATC AAT ATG ATA TCA ATC ATA TGT TTT TTT TTT
**Right_pT_24**	TTT TTT TTT CAT CCA ATA AAT AGT CAA ATC ACC TTT TTT TTT
**Right_pT_26**	TTT TTT TTT TCA TTC CAT ATA TAG TAG CAT TAA TTT TTT TTT
**Right_pT_28**	TTT TTT TTT CAT AAA TAT TCA TGT CTG GAA GTT TTT TTT TTT
**Right_pT_30**	TTT TTT TTT CGG TCA TAG CCC CCT TTT TTC ATT TAT GCG TCA A
**Right_pT_31**	GCG ATT AGC GTC CAA TAC TGC GGA ATC GTT TTT TTT TT
**Right_pT_32**	TTT TTT TTT TGA ATT ACC CGG CAT TTT TTT TTT TTT
**Right_pT_34**	TTT TTT TTT TCA TCA AGA TAA TCA TTG TTT TTT TTT
**Right_pT_36**	TTT TTT TTT GGC AAA AGA GGC TGA CCT TTT TTT TTT
**Right_pT_38**	TTT TTT TTT GGA TCG TCA AAC GAA AGA TTT TTT TTT
**Right_pT_38**	CAG AAA GGC CGC TTT TGC GTT TTT TTT T
**Right_pT_40**	TTT TTT TTT TTT TTT CAC GTT GAA AAT CGG GAG TTC AGC
**Right_pT_42**	TTT TTT TTT TTT CAG GGA TAG TGC GAA TAA TAA TTT TTT TTT
**Right_pT_44**	TTT TTT TTT TTA GCG GGG TTT CAC CAC CCT CAT TTT TTT TTT

**Right_pT_46**	TTT TTT TTT ACA AAC AAA TAA GAA GGA TTA GGA TTT TTT TTT
**Right_pT_48**	GTC ACT TAG CAG CAC CGT AAT CAG TAG CGA CAT TTT TTT TT
**Right_pT_48**	TTT TTT TTT GAA TCA AGT TTG GCC TTG ATA TTC TTT TTT TTT

Table S7. Sequences of connector overhangs c2. Names denote the position of the connector in the folding map in Figure S7, with the line number being where the overhang protrudes from the origami (here: 3').

Connector overhangs c2	
Name	**Sequence 5'→3'**
**c2-0**	6 3 4 7 10<br>TACTAAAGAAATTGCGTAGATTTTCAGGT TTT GTAT GTGTAG TTAGTTAG TGAA AGAGTTG
**c2-8**	CAGAGTCACAAGAGATAGAACCCTTCTGA TTT GTAT GTGTAG TTAGTTAG TGAA AGAGTTG
**c2-18**	AATTGAAGCCGCTACAATTTTATCCGGGAGATTTACAGTAACAGTAC TTT GTAT GTGTAG TTAGTTAG TGAA AGAGTTG
**c2-14**	TTT TAATAACATCACATAATACAT TTT GTAT GTGTAG TTAGTTAG TGAA AGAGTTG
**c2-10**	TTT CCTGAAAGCGTAAACCACCAG TTT GTAT GTGTAG TTAGTTAG TGAA AGAGTTG
**c2-28**	TTT CAGAAGATACTGACCTAAATT TTT GTAT GTGTAG TTAGTTAG TGAA AGAGTTG
**c2-38**	GTCAGGAGAATCCTTTACAGAGAGAACAAAATGTTTCCAGAGCCTAAT TTT GTAT GTGTAG TTAGTTAG TGAA AGAGTTG
**c2-34**	TTT TTAGAATCCTTGTCCCATCCT TTT GTAT GTGTAG TTAGTTAG TGAA AGAGTTG
**c2-30**	TTT TAATGGTTTGAAAAATTCTTA TTT GTAT GTGTAG TTAGTTAG TGAA AGAGTTG
**c2-40**	TTT TTGCCAGTTATAACATAAAAA TTT GTAT GTGTAG TTAGTTAG TGAA AGAGTTG
**c2-46**	TTT AGAGGCATTCAAACGTAGAAA TTT GTAT GTGTAG TTAGTTAG TGAA AGAGTTG
**c2-48**	TTT CCAGTATAAAGGTAAATATTG TTT GTAT GTGTAG TTAGTTAG TGAA AGAGTTG
**c2-4-Cy3**	ACGTAGTAAAAGCAATACTTCTTTGATTAG TTT GTAT GTGTAG TTAGTTAG TGAA AGAGTTG T/Cy3
**c2-44-Cy3**	TTT AATTTACGAGAAACGCAATAA TTT GTAT GTGTAG TTAGTTAG TGAA AGAGTTG T/Cy3
**c2-20-Cy3**	TTT CTTTTACATCTGAATCTTACC TTT GTAT GTGTAG TTAGTTAG TGAA AGAGTTG T/Cy3
**c2-24-Cy3**	TTT TTGAGGATTATTAATTTTCCC TTT GTAT GTGTAG TTAGTTAG TGAA AGAGTTG T/Cy3

Table S8. Sequences of connector overhangs c2*. Names denote the position of the connector in the folding map in Figure S7, with the line number being where the overhang protrudes from the origami (here: 5').

Connector overhangs c2*	
Name	**Sequence 5'→3'**
**c2*-0**	8<br>TCCGAACAAT CC AAC TTT GTGGACTCCAACGTCAAAGGGCGAAAGGAA
**c2*-4**	TCCGAACAAT CC AAC TTT CACACAACATACCCTGGCCCTGAG TTT
**c2*-8**	TCCGAACAAT CC AAC TTT CACTCAATCCGCCAGAATGCGGCG TTT
**c2*-18**	TCCGAACAAT CC AAC TTT GAGTAACAAACTCCAGCC TTT
**c2*-14**	TCCGAACAAT CC AAC TTT AACCTCACCTCATTTGCC TTT
**c2*-10**	TCCGAACAAT CC AAC TTT GAATGACCATAAATCAACAGTTCTGGGCGGTTTC
**c2*-20**	TCCGAACAAT CC AAC TTT ACCCCGGTTGATAATCAGATTTCATCGATT
**c2*-24**	TCCGAACAAT CC AAC TTT CATCCAATAAATAGTCAAATCACC TTT
**c2*-28**	TCCGAACAAT CC AAC TTT CATAAATATTCATGTCTGGAAGTT TTT
**c2*-38**	TCCGAACAAT CC AAC TTT GGATCGTCAAACGAAAGA TTT
**c2*-34**	TCCGAACAAT CC AAC TTT TCATCAAGATAATCATTG TTT
**c2*-30**	TCCGAACAAT CC AAC TTT CGGTCATAGCCCCCTTTTTTCATTTATGCGTCAA
**c2*-40**	TCCGAACAAT CC AAC TTT TTTTTTCACGTTGAAAATCGGGAGTTCAGC
**c2*-44**	TCCGAACAAT CC AAC TTT TTAGCGGGGTTTCACCACCCTCAT TTT
**c2*-46**	TCCGAACAAT CC AAC TTT ACAAACAAATAAGAAGGATTAGGA TTT
**c2*-48**	TCCGAACAAT CC AAC TTT GAATCAAGTTTGGCCTTGATATTC TTT

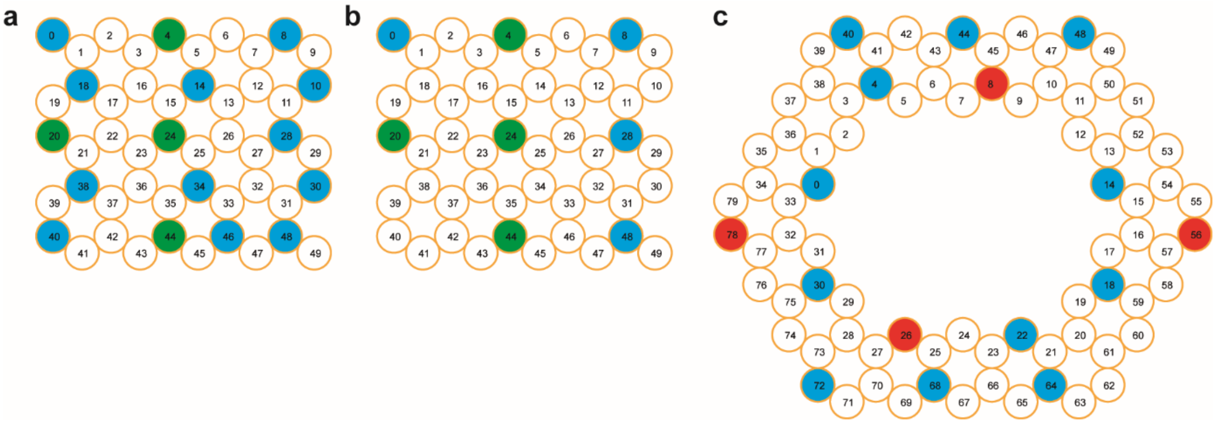

Figure S8. Connector strand positions in the origami. **(a)** Connector strand positions for cuboids with 16 connectors. **(b)** Connector strand positions for cuboids with 8 connectors. **(c)** Connector strand positions for cylinders with 16 connectors. Unmodified connector strands are marked in blue, Cy3 modified connectors in green and Cy5 modified connectors in red.

Table S9. Sequences of free ssDNA strands for the negative feedback loop.

DNA circuit strands	
Name	**Sequence 5’→3’**
**Input**	5* 4* 3* 2* 9*<br>TATTACAC CTAACTAA CTACAC TAATC ATGGAAGATAC
**Activator-IB**	8* 7* 4* 3* 6* 2* 1*<br>ATTGTTCGGA TTCA CTAACTAA CTACAC ATACTTAC TAATC TAGTACTAC /3IAbRQSp/
**IB-Inhibitor-IB**	10* 7* 5* 4* 3* 2* 1*<br>/5IAbRQ/CAACTCT TTCA TATTACAC CTAACTAA CTACAC TAATC TAGTACTAC /3IAbRQSp/
**Fuel A**	8* 10* 7* 4* 3*<br>ATTGTTCGGA CAACTCT TTCA CTAACTAA CTACAC
**Fuel B**	10* 7* 4* 3*<br>CAACTCT TTCA CTAACTAA CTACAC
**Threshold**	6 3 11 3*<br>GTAAGTAT GTGTAG TCTTCT CTACAC
